# A rapid solution-based method for determining the affinity of heroin hapten-induced antibodies to heroin, its metabolites, and other opioids

**DOI:** 10.1007/s00216-018-1060-4

**Published:** 2018-04-19

**Authors:** Oscar B. Torres, Alexander J. Duval, Agnieszka Sulima, Joshua F. G. Antoline, Arthur E. Jacobson, Kenner C. Rice, Carl R. Alving, Gary R. Matyas

**Affiliations:** 1 Laboratory of Adjuvant and Antigen Research, US Military HIV Research Program, Walter Reed Army Institute of Research, 503 Robert Grant Avenue, Silver Spring, MD 20910 USA; 2 U.S. Military HIV Research Program, Henry M. Jackson Foundation for the Advancement of Military Medicine, 6720A Rockledge Drive, Bethesda, MD 20817 USA; 3 Drug Design and Synthesis Section, Molecular Targets and Medications Discovery Branch, Intramural Research Program, National Institute on Drug Abuse and the National Institute on Alcohol Abuse and Alcoholism, National Institutes of Health, Department of Health and Human Services, 9800 Medical Center Drive, Bethesda, MD 20892 USA

**Keywords:** Heroin hapten, Vaccines to substances of abuse, Microscale thermophoresis, ED-UPLC/MS/MS, Apparent dissociation constant (*K*_d_), Binding affinity of unlabeled drug competitors (*K*_i_)

## Abstract

We describe for the first time a method that utilizes microscale thermophoresis (MST) technology to determine polyclonal antibody affinities to small molecules. Using a novel type of heterologous MST, we have accurately measured a solution-based binding affinity of serum antibodies to heroin which was previously impossible with other currently available methods. Moreover, this mismatch approach (i.e., using a cross-reactive hapten tracer) has never been reported in the literature. When compared with equilibrium dialysis combined with ultra-performance liquid chromatography/tandem mass spectrometry (ED-UPLC/MS/MS), this novel MST method yields similar binding affinity values for polyclonal antibodies to the major heroin metabolites 6-AM and morphine. Additionally, we herein report the method of synthesis of this novel cross-reactive hapten, MorHap-acetamide—a useful analog for the study of heroin hapten–antibody interactions. Using heterologous MST, we were able to determine the affinities, down to nanomolar accuracies, of polyclonal antibodies to various abused opioids. While optimizing this method, we further discovered that heroin is protected from serum esterase degradation by the presence of these antibodies in a concentration-dependent manner. Lastly, using affinity data for a number of structurally different opioids, we were able to dissect the moieties that are crucial to antibody binding. The novel MST method that is presented herein can be extended to the analysis of any ligand that is prone to degradation and can be applied not only to the development of vaccines to substances of abuse but also to the analysis of small molecule/protein interactions in the presence of serum.

**Electronic supplementary material:**

The online version of this article (10.1007/s00216-018-1060-4) contains supplementary material, which is available to authorized users.

**Graphical abstract:**
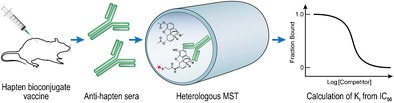
Strategy for the determination of hapten-induced antibody affinities using Microscale thermophoresis

## Introduction

Drug abuse and misuse continue to be at epidemic levels the world over. According to the 2017 World Drug Report, approximately 70% of the global burden of disease resultant of total drug use disorders (29.5 million) was attributable to opioids (~ 20.7 million) [1, 2]. Incidentally, heroin is a drug with one of the highest mortality rates [2]. In the United States alone, the number of deaths from heroin has spiked in the past decade with a 6.2-fold increase from 2002 to 2015 [3], and in October of 2017, the opioid crisis was declared a Public Health Emergency. Among various psychoactive substances, heroin ranks among the worst in terms of the physical harm and strong dependencies that it generates [4]. Therefore, there is an urgent need to develop alternative heroin abuse treatments. Recently, vaccines have been explored as a potential treatment modality for substances of abuse because they do not produce unwanted neurological side effects and they have the potential to be utilized as preventive therapeutics against drug overdose or as synergistic therapies for substance-use disorders [5, 6]. Vaccines to substances of abuse function by generating antibodies that sequester the substance in the blood, thereby preventing it from crossing the blood–brain barrier, engaging its receptor in the brain, and inducing its subsequent psychoactive effects. The primary component of such a vaccine is the hapten–carrier conjugate. In general, substances of abuse are small molecules and consequently do not evoke an immune response by themselves. Thus, an analog (hapten) that structurally mimics the substance is covalently linked to an immunogenic carrier, such as tetanus toxoid (TT), to allow for the substance’s presentation to immune cells [7, 8].

**Fig. 1:**
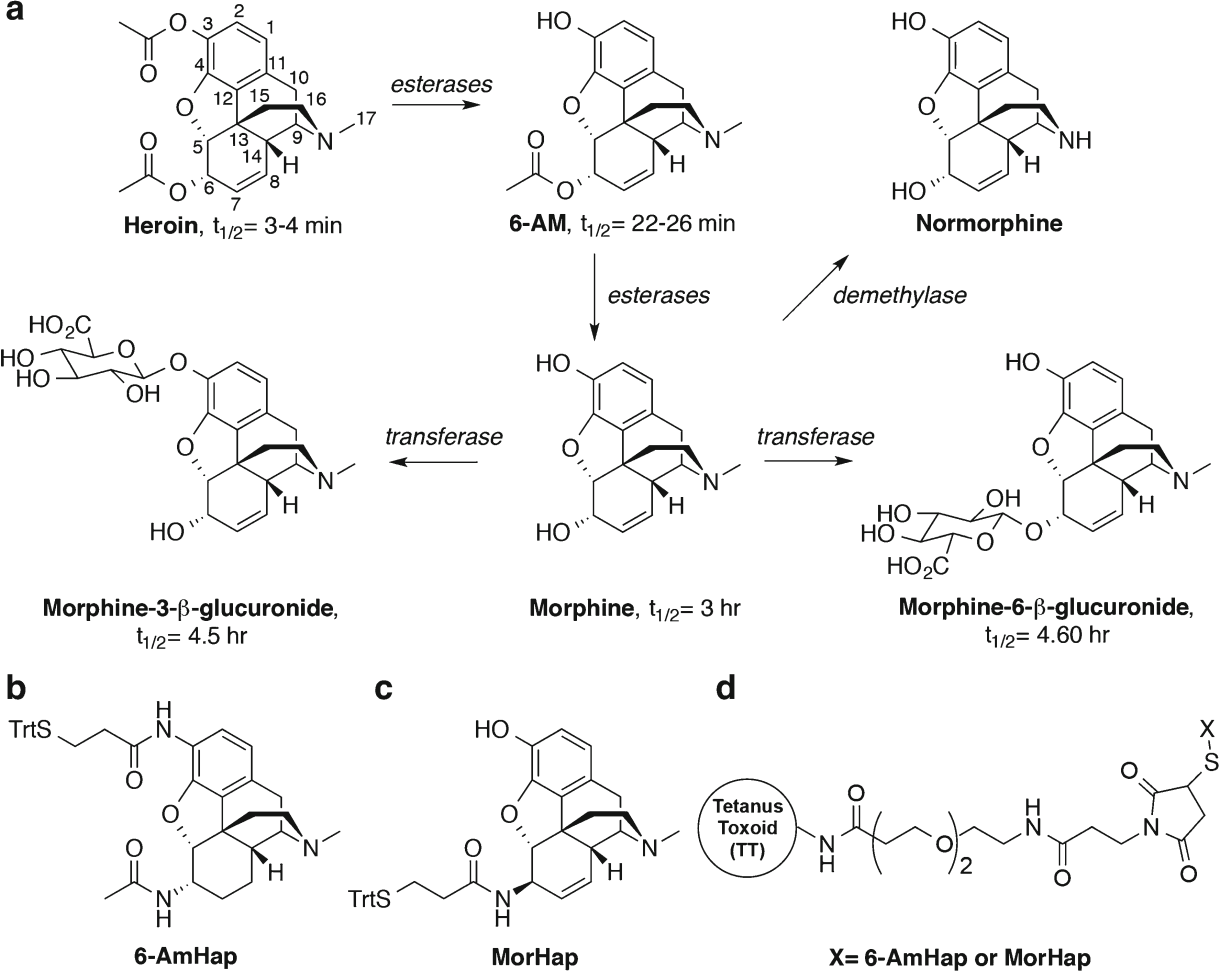
Major heroin metabolites, heroin haptens, and TT–hapten bioconjugates. Degradation of heroin in humans (**a**). C3-linked hapten, 6-AmHap (**b**). C6-linked hapten, MorHap (**c**). Haptens were coupled to tetanus toxoid (TT) to yield the TT–6-AmHap and TT–MorHap conjugates (**d**)

Among such vaccines under development, heroin vaccines are particularly challenging because of the chemical instabilities inherent to heroin’s structure. Heroin is a labile compound with a half-life (*t*_1/2_) of ~ 3–4 min in serum [9]. In vivo, hydrolysis of the C-3 ester by serum esterases generates 6-acetylmorphine (6-AM), and the subsequent hydrolysis of the C-6 ester generates morphine (Fig. 1a). Morphine can then be further metabolized into morphine-6-β-glucuronide (M-6G), which is as neurologically potent as morphine [10], or morphine-3-β-glucuronide (M-3G). To a lesser extent, morphine may also be metabolized into normorphine.

For a heroin vaccine to be effective, the induced antibodies must therefore be able to bind heroin, 6-AM, and morphine [11]. Based on the literature presented above, it may also be beneficial if the induced antibodies can bind M-3G, M-6G, and normorphine in addition. Previously, we conjugated two heroin analogs, 6-AmHap (Fig. 1b) and MorHap (Fig. 1c) to tetanus toxoid yielding vaccine conjugates that abrogated the nociceptive effects of heroin in mice and rats (TT-6-AmHap and TT-MorHap, Fig. **1**d) [12]. Strikingly, 6-AmHap-binding antibodies (6-AmHap-Abs) have a broad range of cross-reactivity to opioids as indicated by the IC_50_ values measured by homologous competition enzyme-linked immunosorbent assay (ELISA). We also developed a method combining equilibrium dialysis with ultra performance liquid chromatography/tandem mass spectrometry (ED-UPLC/MS/MS) to measure the apparent dissociation constant (*K*_d_) of hapten-induced polyclonal antibodies to 6-AM and morphine [13]. Due to its rapid degradation in serum, however, we were unable to use this method (which takes at least 24 h to perform) to measure the *K*_d_ of heroin to polyclonal antibodies. In order to further dissect the cross-reactivity of 6-AmHap-Abs as well as to determine their binding affinity to heroin, we explored other bioanalytical techniques that can measure a solution-based *K*_d_ without requiring long incubation times.

There are several solid-based and solution-based biophysical methods used for the determination of antibody:antigen affinities [14]. Solid-based methods include competition ELISA and surface plasmon resonance (SPR) while solution-based methods include fluorescence quench, isothermal titration calorimetry (ITC), equilibrium dialysis, band-shift, and chromatographic separation. The major drawbacks of solid-based methods are the antigens adsorbed on solid-support may suffer adsorption-induced conformational changes resulting in partial or complete denaturation, and the antigen surface density is hard to predict—potentially affecting the measured antibody binding affinity [15, 16]. As for solution-based methods, both fluorescence quench and ITC require the use of pure antibody and antigen [14] and therefore would not be applicable to the measuring of polyclonal antibody binding in sera. ED in tandem with either radioimmunoassay (RIA) [17, 18] or LC/MS/MS [13] is a useful tool for measuring the binding affinities between polyclonal antibody affinities in sera against small ligands, but is not useful when using labile ligands. Alternatively, antibodies can be labeled with fluorophores or unstable radioactive isotopes that can provide detectable properties [19]. Band-shift and chromatographic separation are standard solution-based methods for measuring antibody:antigen affinity that require these labeled antibodies [19]. There are two main challenges associated using these standard methods, however: non-specific labeling of the antibodies (i.e., label location on the antibody) and the need for extensive purification. As a result, these standard methods typically lead to an unknown amount of inactive antibody. Compounding these problems, we anticipated that band-shift would not be able to discriminate between free antibody and antibody:antigen complexes on native gels due to the small mass of our antigens, whereas chromatographic separation is only applicable for measuring the binding of high affinity antibodies because antibody:small molecule antigen complexes with weak affinity might dissociate prior to or during the chromatographic separation. Lastly, all of these methods, solid- and solution-based, require large quantities of sample [20, 21] making them incompatible with high-throughput experiments when the amount of sera is limited as it often is in mouse studies.

**Fig. 2:**
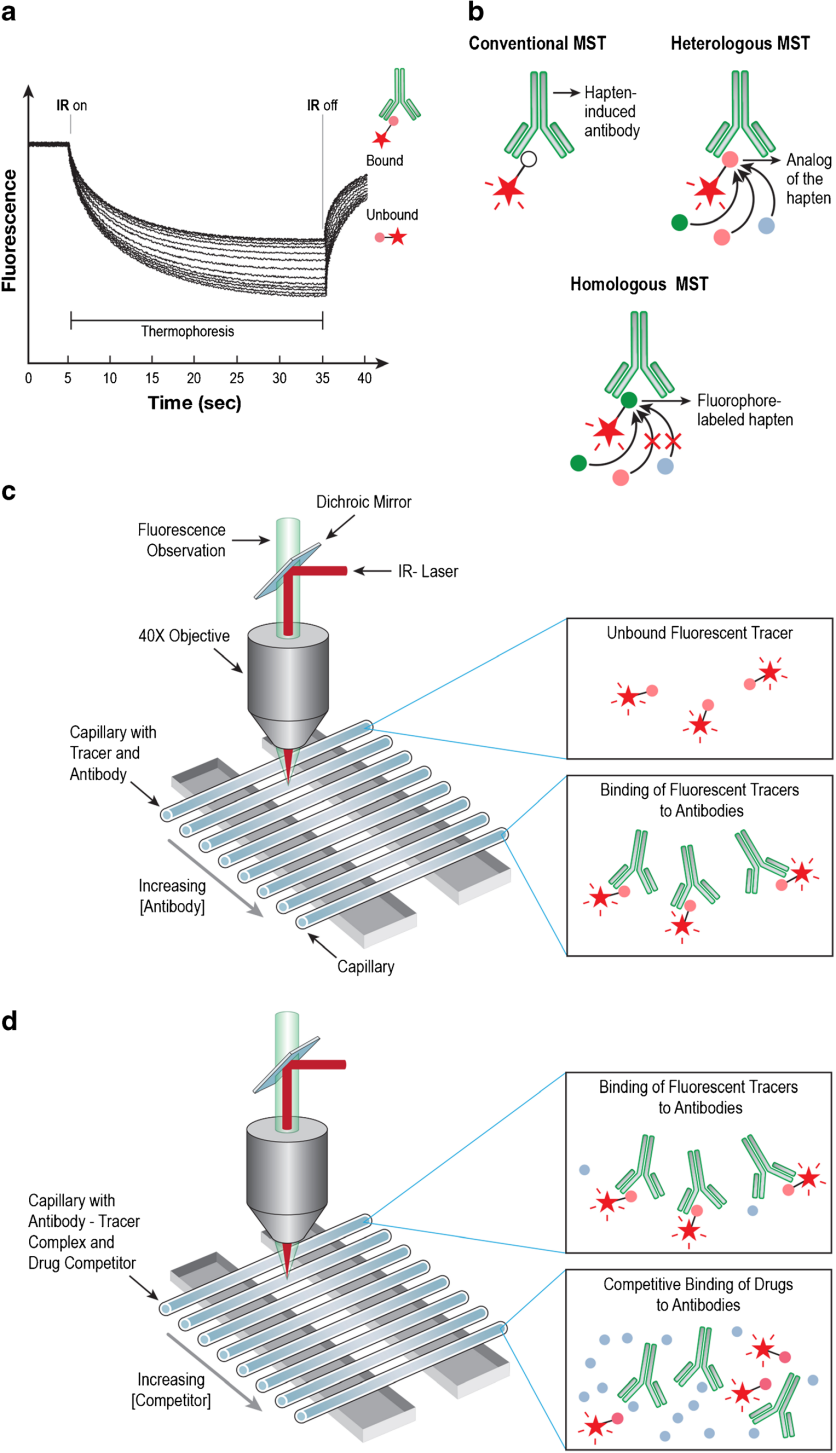
Measurement of antibody binding affinity by MST. Plot of raw fluorescence signal over time for a typical thermophoresis experiment consisting of 16 antibody-drug sample points (**a**). The IR-laser is switched on at *t* = 5 s to induce thermophoresis. In this study, MorHap was conjugated to Cy5 to generate the fluorescent tracer (i.e., MorHap-Cy5). The thermophoretic time trace of antibody-bound fluorescent tracer is different from unbound fluorescent tracer. The IR-laser is switched off at *t* = 35 s, which results in back diffusion. Schematic representation of conventional and competition MST assays (**b**). Since thermophoresis is measured using fluorescence, one of the binding partners must contain a fluorophore. In conventional MST defined herein, the ligand is labeled with a fluorophore. In competition MST, the tracer:antibody complex is competed with various drugs. Homologous competition solely measures the binding affinity of the hapten-induced antibody to the hapten itself. Heterologous competition measures the binding affinity of the hapten-induced antibody to various drugs. Technical setup for the determination of *K*_d_ using conventional MST (**c**). The MST instrument is composed of an IR laser coupled with the path of fluorescence. Excitation/emission detection is through a dichroic mirror. The IR laser is focused via a ×40 objective. The focused IR-laser is used to generate microscopic temperature gradients in an exact spot in the capillary. The thermophoresis is monitored by measuring the changes in fluorescent signal in the same spot. A constant amount of the tracer is titrated with an increasing antibody concentration to generate the saturation binding curve. Technical setup for the determination of IC_50_ using heterologous MST (**d**). A constant amount of the antibody–fluorescent tracer complex is titrated with an increasing concentration of a competitor to generate the competition binding curve and determine the IC_50_. The *K*_d_, IC_50_, and antibody and fluorescent tracer concentrations are used to calculate the *K*_i_

In the last several years, MST, a biophysical technology developed for the analysis of molecular interactions, has received a considerable amount of attention due to its solution-based approach, low sample requirement, and wide range of *K*_d_ measurement capabilities. The technology works on the concept of thermophoresis: the non-Brownian motion of molecules within a temperature gradient [22]. Since an unbound ligand and a ligand:antibody complex have different physical and chemical properties (e.g., size, charge, or solvation shell) and consequently possess different thermophoretic profiles, a binding curve can be constructed and the *K*_d_ can be measured using the thermophoretic data (Fig. 2a). Previously, two major types of MST have been utilized to determine antibody affinity to a ligand: conventional MST and homologous MST (Fig. 2b). In conventional MST, either the antibody [23] or the hapten ligand [22] is fluorescently labeled and titrated with an increasing concentration of the unlabeled binding partner (Fig. 2c). Using this MST data, a *K*_d_ of the antibody to the fluorescently labeled hapten can be measured. In homologous MST, the hapten used to induce the antibody (the natural binding partner) is fluorescently labeled, kept at a constant concentration along with the antibody, and titrated with an increasing concentration of an unlabeled competitor to calculate the 50% inhibition concentration (IC_50_). If the *K*_d_ of the antibody to the fluorescently labeled natural binding partner is known (using conventional MST), the apparent *K*_d_ (*K*_i_) of the antibody to the unlabeled competitor can be calculated indirectly using this and the IC_50_ [16]. For our purposes and for the purposes of the high-throughput development of vaccines to substances of abuse, both of these methods have their drawbacks. First, conventional MST measurements, where the antibody is labeled, require extensive purification. These labeling and purification steps become more complicated when the sample is polyclonal sera [23]. Second, conventional MST measurements, where the ligand is labeled, require the syntheses of several fluorescently labeled ligands if the *K*_d_ for each of ligands is desired. In addition, the *K*_d_ that is being measured is not for the ligand per se, but for the fluorescently labeled ligand. Despite the fact that homologous MST may work equally well for polyclonal antibodies and monoclonal antibodies, a complication is created by the fact that the fluorescently labeled hapten being competed against is the natural binding partner of the antibody. Since any competitor other than the unlabeled natural binding partner itself would likely be unable to out-compete the high affinity complex, homologous MST can only measure the binding affinity of an antibody to a single ligand [16] without potentially skewing any binding affinity data artificially high. The ability, therefore, to accurately measure the affinity of polyclonal antibodies to a large variety of ligands has been an elusive task and has the potential ability to greatly improve upon the methods that are currently used in the analysis of vaccine efficacy. In order to solve the problems presented by the previously described MST methods as well as the other standard methods (vide supra), we herein report a novel type of MST deemed heterologous MST (Fig. 2b). Heterologous MST, much like homologous MST, uses a constant concentration of a fluorescently labeled hapten with a constant concentration of unlabeled antibodies titrated with an increasing concentration of unlabeled competitor in order to indirectly measure the IC_50_ of the antibodies to the competitor (Fig. 2d). Unlike homologous MST, however, heterologous MST uses a fluorescently labeled cross-reactive analog of the natural binding partner that only has a modest affinity to the antibodies. This creates an antibody:fluorescently labeled hapten complex with a much lower affinity, thereby allowing a much broader range of competitors to be tested without artificially skewing the binding affinity data. When conventional MST is used in tandem with heterologous MST to measure the *K*_d_ of the antibodies to the fluorescently labeled hapten, a *K*_i_ can be calculated to a nanomolar accuracy. For instance, a *K*_d_ of polyclonal 6-AmHapAbs to MorHap-Cyanine5 (Cy5), measured using a conventional MST assay, can be used in a heterologous MST assay, using the same 6-AmHapAbs and MorHap-Cy5 but with a morphine competitor, in order to calculate the *K*_i_ of the antibodies to morphine. This method, therefore, makes the task of determining polyclonal antibody binding affinities into a fast and simple tool that can have universal applications for any number of potential competitors.

Unlike ED-UPLC/MS/MS, the only method that could be used previously for similar applications, heterologous MST can be executed in less than 1 h because it does not require the physical isolation of the free and bound ligands or rely on the very slow process of simple diffusion. Because of this reason and the reasons stated vide supra, we hypothesize that heterologous MST in tandem with conventional MST can for the first time feasibly measure the *K*_i_ of polyclonal antibodies (e.g., 6-AmHapAbs) to heroin despite its instabilities. This is the first description of a method capable of reporting the *K*_d_ of antibodies to heroin. Herein, we also demonstrate that (1) *K*_i_ values derived from this tandem conventional/heterologous MST assay are comparable to *K*_d_ values derived from competition ED-UPLC/MS/MS, (2) acetamide-hapten analogs, such as the novel compound MorHap-acetamide, can be useful in studying heroin hapten:antibody interactions, (3) TT-6-AmHap conjugates induce antibodies that cross-react with MorHap and other opioids, and (4) specific structural modifications on the tertiary nitrogen and C-14 position of heroin dramatically reduce the binding of TT-6-AmHapAbs. With the United States recently declaring the opioid crisis to be a National Public Health Emergency, we believe that the analytical method presented in this paper is highly relevant and current. Furthermore, this method has potential far beyond the opioid crisis in immunological studies where metrics other than ELISA titers are required to analyze vaccine efficacy.

## Materials and methods

Sulfo-cyanine5 maleimide (≥ 95%, Cy5) was purchased from Lumiprobe Corporation (Hallandale Beach, Florida, USA). Trifluoroacetic acid (TFA), triethylsilane (Et_3_Si), dimethyl sulfoxide (DMSO), 4-(2-hydroxyethyl)-1-piperazineethanesulfonic acid) (HEPES), triethylamine (Et_3_N), *tert*-butyl-(chloro)diphenylsilane (TBDPCl), imidazole, 2-bromoacetamide, tetrabutylammonium fluoride hydrate (TBAF), cesium fluoride (CsF), chloroform (CHCl_3_), dimethylformamide (DMF), Tween® 20, tetraisopropyl pyrophosphoramide (iso-OMPA), bis(4-nitrophenyl) phosphate (BNPP, 99%), acetanilide (≥ 99.9%), and N-methylpiperidine (99%) were purchased from Sigma-Aldrich (Saint Louis, MO, USA). RediSep® Rf Reversed-phase C18 column/CombiFlash Rf^+^ PurIon Flash chromatography system was purchased from Teledyne Isco (Lincoln, NE, USA). Anti-morphine antibody (ab1060) was purchased from Abcam (Cambridge, MA, USA). Standard treated glass capillary tubes for MST measurements were purchased from NanoTemper Technologies GmbH (Munich, Germany). Drug solutions (1 mg/mL) of 3,6-diacetylmorphine•HCl•H_2_O (heroin•HCl•H_2_O), 6-AM•HCl, morphine•H_2_O, morphine-3-β-glucuronide•H_2_O, morphine-6-β-glucuronide•H_2_O, hydromorphone, naloxone•HCl, normorphine•HCl•H_2_O, nalorphine•HCl, and oxymorphone were purchased from Lipomed (Cambridge, MA, USA). Drug solutions (1 mg/mL) of levorphanol tartrate, desomorphine, thebaine, and meperidine; and drug solutions (100 μg/mL) of morphine N-oxide and 10-hydroxymorphine were purchased from Cerilliant Corporation (Round Rock, TX, USA). All drug solutions were Certified Reference Materials. Optima™ LC/MS grade ammonium formate (NH_4_HCOO), methanol (MeOH), acetonitrile (ACN), and water (H_2_O) were purchased from Fischer Scientific (Suwanee, GA). Waters XBridge® BEH C18 column and screw neck total recovery vial with polytetrafluoroethylene/silicone septa were purchased from Waters (Cambridge, MA, USA). Dulbecco’s phosphate buffered saline (DPBS, 10 mM Na_2_HPO_4_, 1.8 mM KH_2_PO_4_, 2.7 mM KCl, 137 mM NaCl, pH 7.4) was purchased from Quality Biological Inc. (Gaithersburg, MD, USA). All MST experiments were performed in DPBS with 0.05% Tween® 20 (DPBS-Tween).

## Synthesis of MorHap-Cy5

Traditionally, a fluorophore is conjugated to a ligand or protein to generate the fluorescent tracer for MST measurements. It is noteworthy to mention that proteins have intrinsic fluorescence due to tryptophan residues. This intrinsic fluorescence, however, is difficult to utilize when MST measurements are conducted in the presence of dilute serum. Conversely, the intrinsic fluorescence becomes negligible in the presence of a strong fluorophore such as Cy5. In this study, MorHap [6, 12, 24–27], a cross-reactive analog of 6-AmHap, was conjugated to Cy5. The resulting novel fluorescent tracer (MorHap-Cy5) was used in all the MST experiments. 6-AmHap-Cy5 could not be used as a fluorescent tracer because of its high binding affinity to 6-AmHap-Abs (data not shown).

**Fig. 3:**
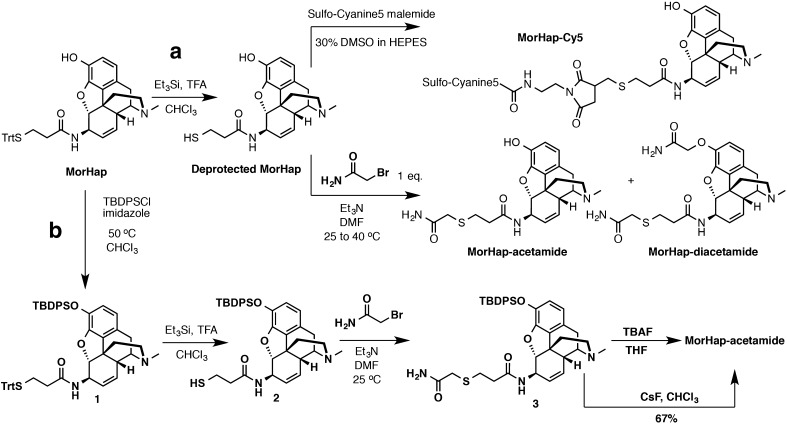
Synthesis of MorHap-Cy5 and MorHap acetamide. Two-step synthesis of MorHap-Cy5 and MorHap acetamide (**a**). Four-step synthesis of MorHap acetamide (**b**). The final step in Scheme *b* did not lead to the formation of MorHap-diacetamide side product

MorHap was synthesized using the procedure of Jalah et al. [26]. MorHap-Cy5 was synthesized from MorHap in two steps (Fig. 3, Scheme *a*). MorHap (35.0 mg, 0.057 mmol) was dissolved in 6 mL chloroform and treated sequentially with TFA (0.44 mL, 57 mmol) and triethylsilane (9.2 μL, 0.057 mmol). The solution was stirred at room temperature (RT) for 30 min and subsequently concentrated at 0 °C on high vacuum for 2 h to produce deprotected MorHap as a white solid. Deprotected MorHap (18.6 mg, 0.05 mmol) was dissolved in a mixture of 30% DMSO in 1 M HEPES buffer (pH 7.0–7.6) (2 mL) and treated with Sulfo-Cyanine5 maleimide (48.2 mg, 0.06 mmol). The reaction mixture was stirred in the dark at RT for 2.5 h. After completion of the reaction, the mixture was injected on a prep scale reverse phase column chromatography system utilizing a RediSep® Rf Reversed-phase C18 column/CombiFlash Rf^+^ PurIon system and a water/acetonitrile (0.1% TFA) mobile phase. Fractions containing MorHap-Cy5 were collected and subsequently lyophilized yielding a dark blue powder (6.87 mg, % yield = 11%, % purity = 97%). HRMS-ESI (*m*/*z*): [M + H]^+^ calculated for C_58_H_69_N_6_O_12_S_3_: 1137.4136; found: 1137.4135.

The purity of the MorHap-Cy5 was assessed by analytical HPLC using the peak area at *A*_630nm_. Analytical HPLC runs of MorHap-Cy5 were carried out on an Agilent 1260 Infinity instrument (Agilent Technologies, Santa Clara, CA, USA) using a Waters XBridge® BEH C18 column, 3.0 × 50 mm, 2.5 μm particle size. The standard gradient was from 5% to 95% acetonitrile in water with 0.1% TFA over 5 min, or from 15% to 50% acetonitrile in water with 0.1% TFA over 5 min. The column flow rate was 0.8 mL/min and column temperature was 30 °C.

The method for the synthesis of the Cy5 negative control tracer is described in the Electronic Supplementary Material (ESM).

## Synthesis of MorHap-acetamide

A novel compound, MorHap-acetamide, was used to measure the binding affinity of monoclonal antibody ab1060 to MorHap, a heroin hapten. The two-step synthesis of MorHap-acetamide was problematic (Fig. 3, Scheme *a*), hence, MorHap-acetamide was synthesized in four steps (Fig. 3, Scheme *b*). The phenol functionality of MorHap was protected using TBDPCl to yield silyl-protected MorHap, **1**. The trityl (Trt) group of MorHap was deprotected using 10% TFA to yield **2**. The free thiol was then reacted with 2-bromoacetamide to yield silyl-protected MorHap-acetamide, **3**. Finally, the silyl-protecting group was removed using CsF or TBAF to yield MorHap-acetamide. The detailed synthesis and analytical characterization of **1**, **2**, **3**, and MorHap-acetamide is described in ESM.

## MST of morphine monoclonal antibody

To confirm that MST can accurately measure antibody binding affinities, the *K*_i_ of morphine to ab1060 was measured. This mouse mAb binds morphine with a manufacturer-reported *K*_d_ value of 2 nM, as determined by a proprietary ultraviolet-visible (UV-VIS) absorption spectroscopy method [28, 29] and by ED-UPLC/MS/MS [13]. Since neither ab1060 nor morphine contain strong fluorophores to allow the direct measurement of binding interactions by MST, and since the presence of a fluorophore on the antibody or the ligand had the potential to produce inaccurate binding data, our strategy was to measure the *K*_i_ in a two-step process. First, the fluorescent tracer MorHap-Cy5 was synthesized and an increasing concentration of ab1060 was used to measure the *K*_d_ to the tracer (conventional MST). Second, in a separate trial, an increasing concentration of morphine was used to displace MorHap-Cy5 in the ab1060:MorHap-Cy5 complex to measure IC_50_ (heterologous MST). The *K*_d_ of ab1060 to the tracer and the IC_50_ of morphine to the complex were used together to calculate the *K*_i_ of ab1060 to morphine (vide infra).

The manufacturer reported that the ab1060 standard was generated using a BSA–morphine analog conjugate. This conjugate was synthesized by conjugating BSA to the hydroxy group at the C-6 position of the morphine analog through a linker [28]. Although the structure of the morphine analog is proprietary, it can be inferred that it has a similar structure to MorHap. Therefore, ab1060 should be able to recognize MorHap-Cy5 and MorHap-acetamide.

### Conventional MST

The binding affinities of ab1060 to MorHap-Cy5 and Cy5 (negative control) were determined at 0.25, 0.5, and 1.0 nM tracer concentrations using conventional MST. Different MorHap-Cy5 concentrations were used to test if the concentration of the tracer has a significant effect on the *K*_d_ measurement. For the 0.25 nM MorHap-Cy5 assay condition, working solutions of ab1060 (6400 nM) and MorHap-Cy5 (0.5 nM) were prepared in DPBS-Tween. The working solution of ab1060 (10 μL) was serially diluted (1:1) in 200 μL vials each containing DPBS-Tween (10 μL) to yield 16 different antibody concentrations, the highest of which was 3200 nM. Each 10 μL volume of ab1060 was subsequently mixed with an equal volume of the tracer (10 μL) and incubated at RT in the dark for 20 min. The MorHap-Cy5:ab1060 mixtures were then loaded in capillary tubes for MST measurements. The same procedure was used to measure the binding affinity of ab1060 at 0.5 nM and 1.0 nM tracer concentrations except that the stock solutions had different concentrations.

### Heterologous MST

The binding affinity of ab1060 to morphine was determined at 15 different assay conditions utilizing three different MorHap-Cy5 concentrations (0.25, 0.5, and 1.0 nM) and five different ab1060 concentrations (5.0, 10.0, 20.0, 30.0, and 40.0 nM). The varying concentrations of MorHap-Cy5 and ab1060 were used to test if there are optimal conditions for measuring *K*_i_ or if there is a tolerance for variation. For a 0.25 nM MorHap-Cy5/10.0 nM ab1060/4000 nM morphine heterologous assay condition, working solutions of ab1060 (40.0 nM), tracer (1.0 nM), and morphine (16,000 nM) were prepared in DPBS-Tween. Equal parts of ab1060 and MorHap-Cy5 working solutions were mixed together and incubated at RT in the dark for 20 min. The working solution of morphine (10 μL) was serially diluted (1:1) in 200-μL vials each containing DPBS-Tween (10 μL) to yield 16 different concentrations, the highest of which was 8000 nM. The ab1060:MorHap-Cy5 mixture was mixed in equal volume (10 μL) to each tube of diluted morphine and incubated at RT in the dark for 20 min. The ab1060:MorHap-Cy5:morphine mixtures were then loaded in capillary tubes for MST measurements.

The same procedure was performed for the other 14 assay conditions but with different concentrations of MorHap-Cy5 and ab1060 working solutions. Working solutions of 2.0 nM and 4.0 nM tracer were used for assay conditions of 0.5 nM and 1.0 nM MorHap-Cy5, respectively. Working solutions of 20.0, 80.0, 120.0, and 160.0 nM antibody were used for assay conditions of 5.0, 20.0, 30.0, and 40.0 nM ab1060, respectively. The binding affinity of ab1060 to MorHap-acetamide was determined at a 0.25 nM MorHap-Cy5/10.0 nM ab1060/4000 nM MorHap-acetamide assay condition as described above.

## Determination of *K*_d_ and antibody binding site concentration of polyclonal 6-AmHap-abs using ED-UPLC/MS/MS

Post-immune sera (week 8) from the mouse study described by Sulima et al. [12] were used in this study. The *K*_d_s of 6-AmHap-Abs to 6-AM and morphine, which were subsequently used to approximate the concentration of antibody binding sites, were determined by ED-UPLC/MS/MS in accordance with the method outlined by Torres et al. [13]. Briefly, post-immune serum was diluted in DPBS (1:1600). Dialysis buffer was prepared by diluting the corresponding pre-immune serum (week 0) in DPBS (1:1600). Competitive inhibitors, 6-AM or morphine, were prepared in the dialysis buffer. A 100-μL dilution of week 8 serum containing 5 nM D_3_-tracer was pipetted into the sample chamber, and a 300-μL aliquot of the competitive inhibitor solution was added to the buffer chamber. Both week 8 and week 0 were run against each other at the same sera dilutions. For negative controls, the same setup was employed except both sides of the dialysis chamber contained only week 0 serum. Equilibrium dialysis, preparation of samples for UPLC/MS/MS, and calculation of *K*_d_ were performed as described [13].

The antibody binding site concentration was derived from Müller’s equation [17] and was calculated using the following formula:

$$ \left[{Ab}_t\right]=b\left[{T}_t\right]+\frac{b\times {K}_d}{\left(1-b\right)} $$

where [*Ab*_*t*_] is the total concentration of the antibody binding sites, *b* is the fraction of bound D_3_-tracer in the absence of competitive inhibitor, [*T*_*t*_] is the total molar concentration of the D_3_-tracer after equilibrium, and *K*_d_ is the apparent dissociation constant. The concentration of 6-AmHap-Abs ([6-AmHap-Abs]) was calculated by averaging the number of antibody binding sites for 6-AM and morphine in the polyclonal mouse serum.

## Heroin degradation in sera

To accurately determine the binding affinity of polyclonal serum to heroin, heroin degradation must be suppressed during the execution of the heterologous MST assay. Since competition MST is performed at a constant concentration of antibody and a varying concentration of drug competitor, the heroin degradation studies were performed with a constant dilution of serum and a varying concentration of heroin to mimic the heterologous MST assay conditions.

Heroin degradation was monitored in DPBS, dilute pre-immune serum (week 0), and dilute post-immune serum (week 8) containing 6-AmHap-Abs. A 400-μL aliquot of heroin at 5.0, 10.0, 20.0, or 4000 nM in DPBS was treated with a 1:200 dilution of mouse serum. These heroin concentrations were used because 5.0, 10.0, and 20.0 nM were equivalent, per ED-UPLC/MS/MS, to half, equal, and twice, respectively, the concentration of the antibody binding sites in this dilution, as well as in the other polyclonal heterologous MST assays. Furthermore, 4000 nM was used because it was the highest concentration of heroin used in the heterologous MST assays (vide supra). The reaction mixture was incubated at RT and the amount of remaining heroin was monitored at given time intervals over 3 h using UPLC/MS/MS. The details of the UPLC/MS/MS conditions for measuring the concentration of heroin were previously described [13]. The amount of heroin was expressed as a percent heroin (% Heroin) and was calculated using the following equation:

$$ \% Heroin=\frac{{\left[ Heroin\right]}_t}{{\left[ Heroin\right]}_{initial}}\times 100 $$

where [Heroin]_*t*_ is the concentration of the heroin at time *t* and [Heroin]_initial_ is the initial concentration of heroin.

Heroin degradation was also monitored in the presence of the esterase inhibitors iso-OMPA and BNPP. Aliquots (400 μL) of both the pre-immune and post-immune sera were pre-treated with 20 μM iso-OMPA/20 μM BNPP for 2 h at RT. Heroin (4000 nM) was added to the reaction mixtures and the % Heroin was monitored as described above.

The uninhibited degradation curve of heroin was fitted using the non-linear regression one-phase decay method in GraphPad Prism version 7.0a. The half-life (*t*_1/2_) of heroin was determined with a 99% confidence interval and was reported as a range. The goodness of the fit of the curve was reported as *r*^2^.

## MST of polyclonal 6-AmHap-abs

### Conventional MST

The binding affinity of 6-AmHap-Abs to MorHap-Cy5 and Cy5 (negative control) was determined at a 0.25 nM tracer concentration and a 50 nM starting antibody concentration. A working solution of post-immune serum (~ 1:12 dilution, or about 200 nM antibody binding site concentration) containing 6-AmHap-Abs was prepared in DPBS-Tween. The serum working solution was serially diluted in 200-μL vials each containing DPBS-Tween (10 μL) to yield 16 different antibody binding site concentrations, the highest of which was 100 nM. Each dilution of serum (10 μL) was then mixed with an equal volume of MorHap-Cy5 and incubated at RT in the dark for 20 min. The 6-AmHap-Abs:MorHap-Cy5 mixtures were then loaded in capillary tubes for MST measurements.

### Heterologous MST

The binding affinities of 6-AmHap-Abs to heroin, its metabolites, and other unlabeled opioids were determined at fixed MorHap-Cy5 (0.25 nM) and antibody binding site (10 nM) concentrations. The binding affinity of 6-AmHap-Abs to heroin was measured both in the absence and in the presence of esterase inhibitors. For the MST heroin-binding assay in the absence of esterase inhibitors, working solutions of 6-AmHap-Abs (40 nM), MorHap-Cy5 (1 nM), and heroin (16,000 nM) were prepared in DPBS-Tween. Equal parts 6-AmHap antibody and MorHap-Cy5 working solutions were mixed together and incubated at RT in the dark for 20 min. The working solution of heroin (5 μL) was serially diluted (1:1) in 200-μL vials each containing DPBS–Tween (5 μL) to yield 16 different concentrations, the highest of which was 8000 nM. The 6-AmHap-Abs:MorHap-Cy5 mixture was then mixed in equal volume (5 μL) to each tube of diluted heroin and incubated at RT in the dark for 20 min. The 6-AmHap-Abs:MorHap-Cy5:morphine mixtures were then loaded in capillary tubes for MST measurements. The final concentrations of 6-AmHap-Abs, MorHap-Cy5 in the capillaries were 10 nM and 0.25 nM, respectively. The same procedure was performed for the binding of the 6-AmHap-Abs to the other drugs but different concentrations of competitor working solutions were used. For example, starting concentrations of 25,000, 50,000, and 100,000 nM were used for normorphine, morphine N-oxide, and naloxone, respectively.

For the heroin-binding assay in the presence of esterase inhibitors, a working solution containing 6-AmHap antibody (40 nM) and 80 μM iso-OMPA/80 μM BNPP was incubated for 2 h at RT. An equal volume of MorHap-Cy5 (1 nM) was then mixed with the 6-AmHap-Abs/iso-OMPA/BNPP mixture and the reaction mixture was further incubated for 20 min in the dark at RT. The rest of the procedure was performed as described above. The final concentrations of 6-AmHap-Abs, iso-OMPA/BNPP, and MorHap-Cy5 in the capillaries were 10 nM, 20 μM/20 μM, and 0.25 nM, respectively.

## MST measurements and calculation of apparent dissociation constants

The MST measurements were done on a Monolith™ NT.115^Pico^ instrument from NanoTemper Technologies GmbH. The instrument was equipped with an IR laser (wavelength, 1475 ± 15 nm; power, 120 mW maximum) and a red fluorescence channel that can detect red dyes, such as Cy5 and Alexa Fluor 647. The samples were measured at an LED power of 20% and an MST power of 25% with a laser-on time of 30 s and a laser-off time of 5 s. MST measurements of 15–16 samples typically take ~ 20 min.

In the conventional MST procedures, a constant concentration of MorHap-Cy5 was titrated with an increasing concentration of antibody. The *K*_d_ values of MorHap-Cy5 against ab1060 and 6-AmHap-Abs were derived using MO.Affinity Analysis v2.2.4* software. The detailed mathematical derivation of the *K*_d_ using MST is discussed elsewhere [22, 30, 31]. Briefly, the software calculates the normalized fluorescence (*F*_norm_) of the tracer at a given antibody concentration, using the following equation:

$$ {F}_{norm}=\frac{F_h}{F_c}\ast 1000 $$

where *F*_norm_ is related to the concentration of free tracer and antibody-bound tracer and expressed as a permille (^0^/_00_). “Cold fluorescence” (*F*_c_) is the mean steady-state fluorescence before the IR laser is on while “hot fluorescence” (*F*_h_) is the mean steady-state fluorescence at a point after the IR-laser is on. The point with the highest sound-to-noise ratio and *K*_d_ confidence was manually selected as *F*_h_. The software plots *F*_norm_ against the increasing 15–16 antibody concentration points to yield the binding curve, and subsequently fits the curve to obtain the *K*_d_. The software also plots the fraction of tracer that is bound to the antibody (Fraction Bound) against increasing antibody concentration, using the following equation:$$ {\displaystyle \begin{array}{c}\boldsymbol{Fraction}\ \boldsymbol{bound}=\frac{\boldsymbol{x}-{\boldsymbol{F}}_{\boldsymbol{\operatorname{norm}},\boldsymbol{Unbound}}}{\boldsymbol{Response}\ \boldsymbol{amplitude}}\\ {}\boldsymbol{Response}\ \boldsymbol{amplitude}={\boldsymbol{F}}_{\boldsymbol{\operatorname{norm}},\boldsymbol{Bound}}-{\boldsymbol{F}}_{\boldsymbol{\operatorname{norm}},\boldsymbol{Unbound}}\end{array}} $$

where *x* is the *F*_norm_ at a given antibody concentration, *F*_norm,Unbound_ is the lowest *F*_norm_ plateau value, and *F*_norm,Bound_ is the highest *F*_norm_ plateau value.

In the competition MST procedures, a constant concentration of MorHap-Cy5:antibody complex was titrated with an increasing concentration of drug competitor. The IC_50_ values of competitor drugs against ab1060 and 6-AmHap-Abs were derived using the same affinity analysis software. The software plots *F*_norm_ against 15–16 increasing drug competitor concentration points to yield the binding curve, and subsequently fits the curve to obtain the IC_50_. For presentation purposes, the fraction bound was manually calculated and plotted using the equation above where *x* is the *F*_norm_ at a given competitor concentration. The binding affinities (*K*_i_) from the heterologous MST assays were then derived using the K_i_-Finder software from the Nanotemper website [32]. In order to calculate the *K*_i_, the following parameters must be entered into the K_i_-Finder website: the binding affinity of the tracer against the antibody (*K*_d_), the concentration of the antibody (*I*_0_), the concentration of the tracer (labeled *T*_0_), the highest concentration of the competitor (*L*_0_), and the IC_50_ of the competitor to the antibody. The website then plots both the binding curve and competition curve to produce the competitor *K*_i_, also known as the *K*_d_ of the antibody to the drug competitor.

As the MST signal is sensitive to an abrupt buffer composition change, the highest titrant concentration point for all MST assays was on occasion removed for the purposes of calculating the IC_50_ or *K*_d_. For instance, for the conventional MST of polyclonal serum, the experiment was performed starting at a 6-AmHap-Abs concentration of 100 nM (i.e., low serum dilution). However, the 100 nM concentration point was removed in the calculation of *K*_d_ because the baseline fluorescence and bleaching rates of the sera at 100 nM was both higher than the recommended value and resulted in artifactual signal.

## Data analysis

Statistical analyses were performed using GraphPad Prism version 7.0a. A one-way analysis of variance (ANOVA), Kruskal–Wallis test with Dunn’s correction for multiple comparisons was used to compare the *K*_d_ values of ab1060 to MorHap-Cy5 with different fluorescent tracer concentrations, the *K*_i_ values of ab1060 to morphine derived from the 15 various assay conditions, and the *K*_i_ values of ab1060 to morphine derived from UV-VIS, MST, and ED-UPLC/MS/MS. A Mann–Whitney non-parametric *T* test was used to compare the *K*_i_ values of 6-AmHap-Abs to heroin in the presence and absence of esterase inhibitors. A Mann–Whitney non-parametric *T* test was also used to compare the binding affinities of 6-AmHap-Abs to 6-AM and morphine derived from MST and ED-UPLC/MS/MS. The binding curves were re-plotted using GraphPad Prism version 7.0a for presentation purposes.

## Results

### Synthesis of MorHap-Cy5 and MorHap-acetamide

The deprotection of the thiol followed by treatment with the Sulfo-Cyanine5 maleimide in HEPES buffer afforded the desired product. Subsequent purification by reverse phase chromatography gave MorHap-Cy5 in 11% yield over two steps. Conversely, the synthesis of MorHap-acetamide using Scheme *a* was a challenge (Fig. 3). The deprotection of the thiol again proceeded smoothly, but the subsequent alkylation was non-selective. A mixture of unidentified mono- and dialkylated products was detected (Fig. 3, Scheme *a*). To solve this, the phenol MorHap was protected by the *tert*-butyl diphenylsilyl ether (TBDPSCl) in order to facilitate survival during the acidic Trt cleavage conditions (Fig. 3, Scheme *b*), forming **1**. Alkylation of **2** with 2-bromoacetamide gave amide **3** in 64% yield. TBAF deprotection of amide **3** proceeded smoothly to produce the phenolic product, MorHap-acetamide. Unfortunately, during purification via reversed-phase chromatography, the TBAF salts co-eluted with the desired product. The simple workaround was to switch from TBAF to cesium fluoride, which afforded the desired MorHap-acetamide in 67% yield.

### MST of morphine monoclonal antibody

**Fig. 4:**
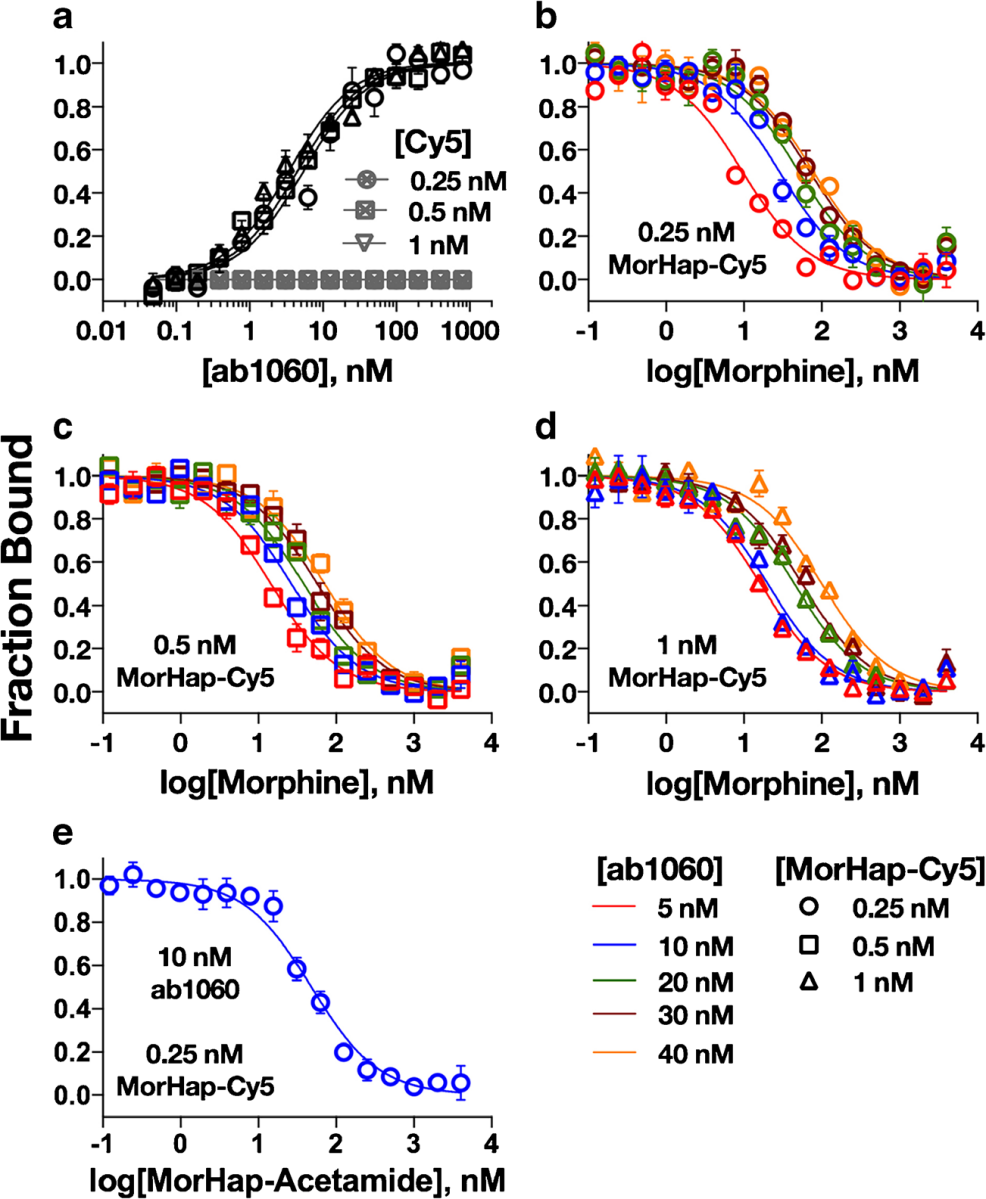
Saturation and competition binding curves for ab1060 monoclonal antibody. Saturation binding curve (conventional MST) of ab1060 against different concentrations of MorHap-Cy5 and Cy5, negative control (**a**). Competition binding curves of ab1060 against morphine at different ab1060 concentrations and at fixed MorHap-Cy5 concentrations: 0.25 nM (**b**), 0.5 nM (**c**), and 1 nM (**d**). Competition binding curve of ab1060 against MorHap-acetamide at 10 nM ab1060/0.25 nM MorHap-Cy5 concentration (**e**). The binding curves are derived from three independent experiments ± standard error of the mean

The *K*_d_ values of ab1060 against 0.25, 0.5, and 1.0 nM MorHap-Cy5 were 5.29 ± 1.67 nM, 4.25 ± 1.34 nM, and 2.95 ± 1.93 nM, respectively (Fig. 4a, black lines). There were no significant differences in *K*_d_ values obtained at different tracer concentrations (*p* = not significant, one-way ANOVA). The *K*_d_ values from all of the trials of the three different MorHap-Cy5 concentrations were therefore averaged (*K*_d_ = 4.58 ± 2.19 nM) using the MO.Affinity Analysis software. This value was subsequently used to calculate the *K*_i_ of ab1060 to morphine and MorHap-acetamide (see below). Ab1060 did not, however, bind the Cy5 fragment of the tracer (Fig. 4a, gray lines).

At 0.25 nM MorHap-Cy5 and varying antibody concentrations, the IC_50_ of ab1060 to morphine increased commensurately with antibody concentrations (Fig. 4b, see ESM Table S1). The same trend was observed for both 0.5 nM (Fig. 4c) and 1.0 nM (Fig. 4d) MorHap-Cy5 concentrations. The *K*_i_ values of ab1060 to morphine at different morphine and antibody concentrations (see ESM Table S1) were not statistically different from each other (*p* = not significant, one-way ANOVA). In addition, these *K*_i_ values for ab1060:morphine were not statistically different from the *K*_d_ values derived by ED-UPLC/MS/MS (*K*_d_ = 1.97 ± 0.17 nM) or from the *K*_d_ values derived by the manufacturer with UV-VIS (*K*_d_ = 2 nM). Since the different combinations of tracer and antibody concentrations do not have a statistically significant impact on *K*_i_ values, the heterologous competition of ab1060 with MorHap-acetamide was calculated at 0.25 nM tracer and 10 nM antibody concentrations. These conditions were also used for the determination of the *K*_i_ value of polyclonal serum to all other opioids (vide supra). The *K*_i_ value of ab1060 to MorHap-acetamide was 13.99 ± 7.97 nM (Fig. 4e).

### Heroin degradation in serum

**Fig. 5:**
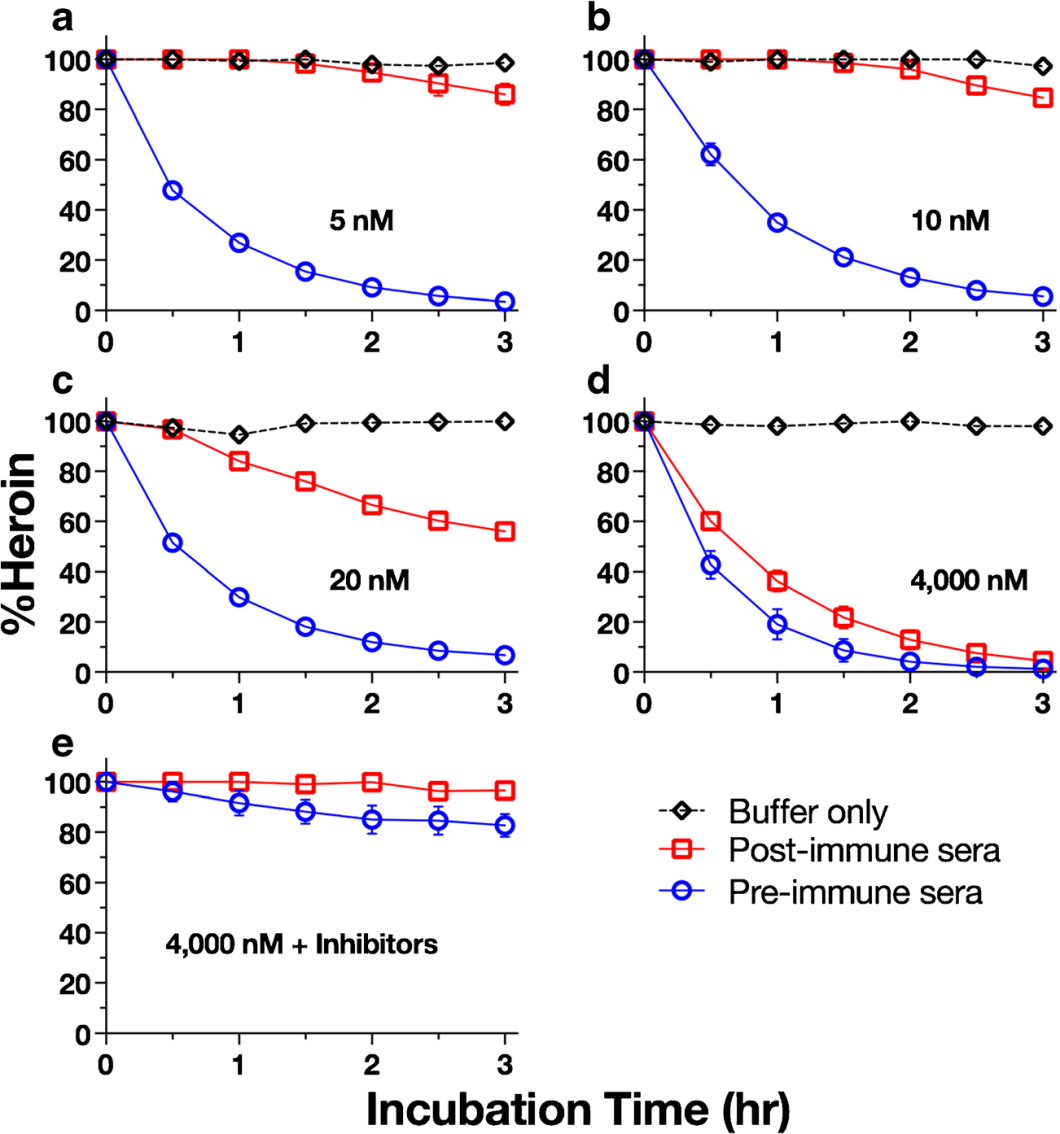
Degradation profile of heroin in buffer, and in post-immune and pre-immune sera. Starting heroin concentrations of 5 nM (**a**), 10 nM (**b**), 20 nM (**c**), 4000 nM without inhibitors (**d**), and 4000 nM with esterase inhibitors (**e**) were used in the assay. Heroin was quantified by UPLC/MS/MS. % Heroin is derived from three independent experiments ± standard error of the mean

The degradation of heroin at different concentrations was monitored both in the presence and absence of dilute sera (Fig. 5a–e). Both pre-immune and post-immune sera contain esterases, though only post-immune serum contains 6-AmHap-Abs (~ 10 nM). In the absence of serum (i.e., buffer alone), no heroin degradation was observed over the course of 3 h at 5, 10, 20, or 4000 nM heroin concentrations. In the presence of dilute pre-immune serum, however, extensive degradation of heroin was observed after 1 h (% Heroin = 27.7 ± 8.0%), and almost complete degradation was observed after 3 h (% Heroin = 4.3 ± 2.5%) regardless of the starting heroin concentration. In addition, the degradation of heroin in pre-immune serum seemed to follow first-order kinetics as judged by the goodness of fit of the degradation curve (5 nM, *r*^2^ = 0.996; 10 nM, *r*^2^ = 0.990; 20 nM, *r*^2^ = 0.997; 4000 nM, *r*^2^ = 0.975). The half-lives of heroin in the presence of pre-immune serum were comparable regardless of the starting heroin concentration (5 nM, *t*_1/2_ = 0.417–0.541 h; 10 nM, *t*_1/2_ = 0.555–0.832 h; 20 nM, *t*_1/2_ = 0.449–0.556 h; 4000 nM, *t*_1/2_ = 0.305–0.545 h). Interestingly however, we found that the degradation of heroin in the presence of post-immune serum was entirely dependent on the starting heroin concentration. No significant degradation of heroin was observed over the course of 3 h when the starting heroin concentration was less than or equal to the concentration of the 6-AmHap binding sites (10 nM) (Fig. 5a, b). When the starting heroin concentration was twice (20 nM, Fig. 5c) the 6-AmHap binding site concentration, only a small amount of heroin was degraded after 1 h (% Heroin = 84.1 ± 3.4%) but nearly half of the heroin was degraded after 3 h (% Heroin = 56.1 ± 3.3%). In the presence of post-immune serum, the degradation of heroin did not follow first order kinetics (5 nM, *r*^2^ = 0.553, *t*_1/2_ = undefined; 10 nM, *r*^2^ = 0.725, *t*_1/2_ = undefined; 20 nM, *r*^2^ = 0.967, *t*_1/2_ = undefined). When the starting heroin concentration was 400 times that of the 6-AmHap binding site concentration (4000 nM, Fig. 5d), extensive degradation of heroin was observed after 1 h. At 4000 nM heroin, both dilute post-immune and pre-immune sera exhibited similar degradation profiles. The degradation of 4000 nM heroin in the presence of post-immune serum followed first order kinetics with *r*^2^ = 0.981 and *t*_1/2_ = 0.527–0.947 h. In the presence of esterase inhibitors BNPP and iso-OMPA, however, the degradation of heroin was drastically reduced in dilute pre-immune serum (% Heroin = 91.76 ± 8.64%) and completely abolished in dilute post-immune serum (% Heroin = 100%) at the 1 h time point (Fig. 5e). Under these conditions, the degradation of heroin in both dilute post-immune (*r*^2^ = 0.349, *t*_1/2_ = undefined) and pre-immune sera (*r*^2^ = 0.455, *t*_1/2_ = undefined) did not follow first-order kinetics.

### MST of polyclonal 6-AmHap-abs

**Fig. 6:**
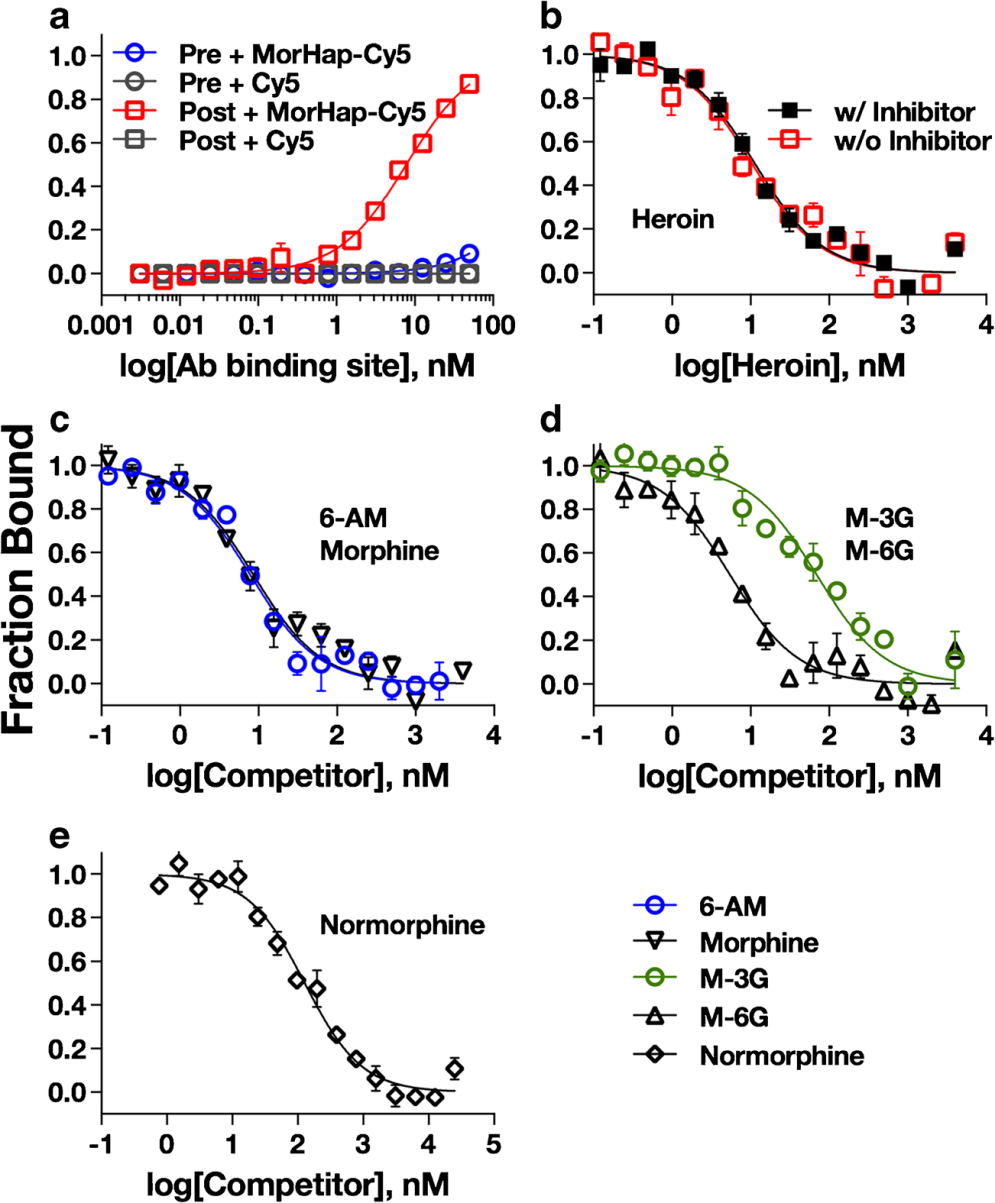
Saturation and competition binding curves for 6-AmHap-Abs. Saturation binding curves of pre-immune (Pre) and post-immune (Post) sera against MorHap-Cy5 and Cy5, negative control (**a**). Post-immune serum contains 6-AmHap-Abs. Competition binding curves of 6-AmHap-Abs against heroin with and without inhibitors (**b**); major heroin metabolites, 6-AM and morphine (**c**); downstream heroin metabolites, M-3G, M-6G (**d**); and normorphine (**e**). The binding curves are derived from three independent experiments ± standard error of the mean. Values are the mean of triplicate determinations ± standard deviation

The binding affinities of post-immune sera (week 8) containing 6-AmHap-Abs, and pre-immune (week 0) against MorHap-Cy5 and Cy5 (negative control) were measured using conventional MST (Fig. 6a). Pre-immune serum did not bind either MorHap-Cy5 (blue circle) or Cy5 (gray circle). In contrast, antibodies in the post-immune serum did bind MorHap-Cy5 (*K*_d_ = 7.65 ± 0.87 nM, red square), but did not bind Cy5 (gray square).

The binding of 6-AmHap-Abs to heroin was determined with and without esterase inhibitors (Fig. 6b). The 6-AmHap-Abs tightly bound heroin both with inhibitors (*K*_i_ = 1.60 ± 0.75 nM, filled black square) and without inhibitors (*K*_i_ = 2.27 ± 1.47 nM, red square). There was no significant difference between the two *K*_i_ values (*p* = not significant, *T* test). The binding of 6-AmHap-Abs to heroin metabolites was also measured. The 6-AmHap-Abs tightly bound the primary heroin metabolites, such as 6-AM (*K*_i_ = 0.44 ± 0.33 nM) and morphine (*K*_i_ = 1.00 ± 0.66 nM) (Fig. 6c). To establish that the MST method can be used to measure the affinities of antibodies induced by a TT–hapten bionconjugate to various drugs, the binding affinities of 6-AmHap-Abs were also determined by ED-UPLC/MS/MS (ESM Table S2). The *K*_d_s of 6-AmHap-Abs to 6-AM (*K*_d_ = 0.53 ± 0.28 nM) and morphine (*K*_d_ = 0.51 ± 0.19 nM) that were derived using ED-UPLC/MS/MS were not statistically significantly different from the *K*_i_s derived from MST (*p* = not significant, *T* test). These results suggest that *K*_d_ can be calculated to a high degree of accuracy at greater than 1:50 serum dilution using MST.

**Fig. 7:**
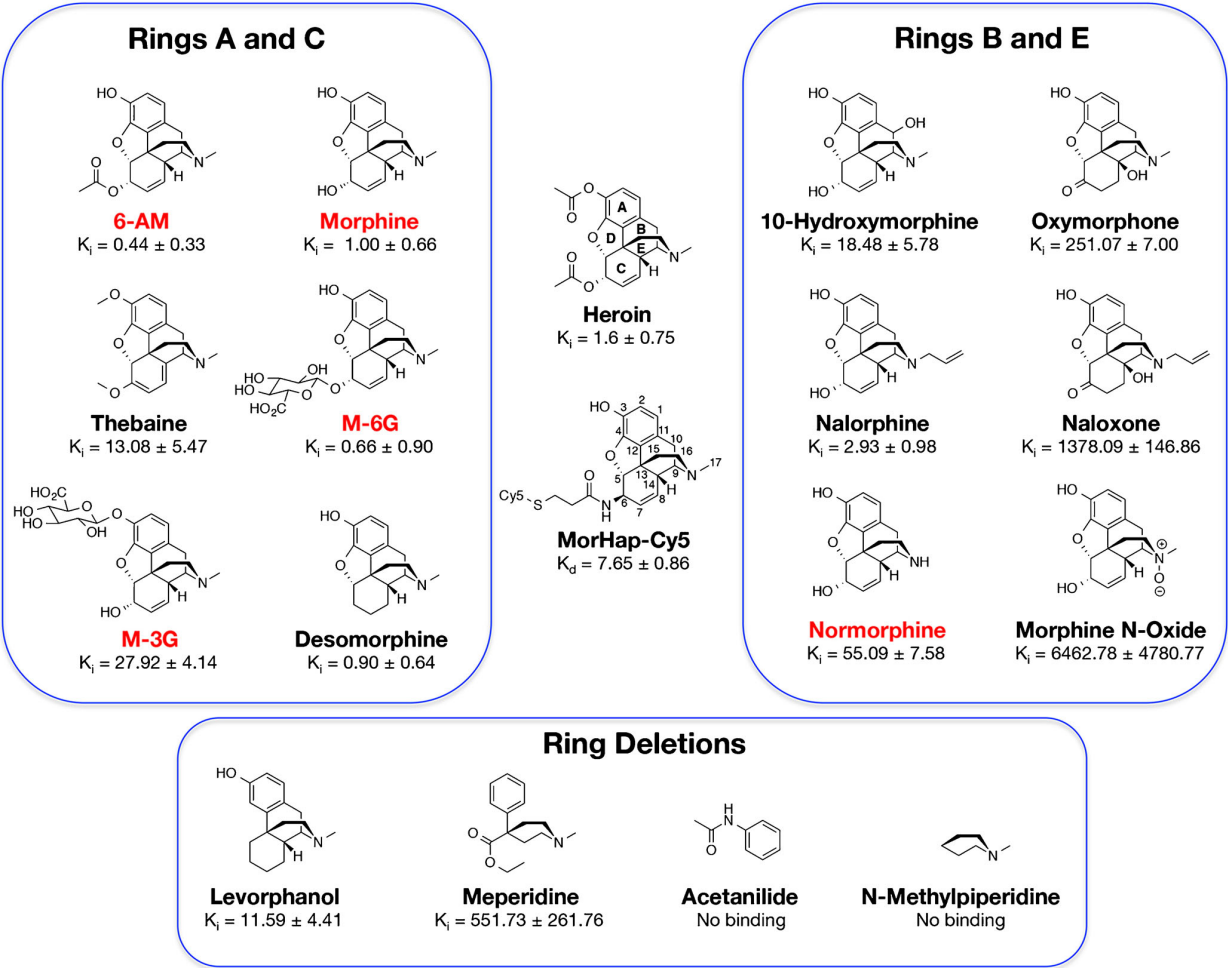
Binding affinity (*K*_i_) values of various opioids to 6-AmHap-Abs. The main modifications to heroin’s structures are classified in terms of rings A and C, and rings B and E modifications, as well as ring deletions. Heroin metabolites are shown in red. Values are reported in nanomolar and are derived from three independent experiments ± standard deviation

The 6-AmHap-Abs also bound the downstream metabolites: morphine-3-β-glucuronide (*K*_i_ = 27.92 ± 4.14 nM) and morphine-6-β-glucuronide (*K*_i_ = 0.66 ± 0.90 nM) (Fig. 6d), and the minor metabolite, normorphine (*K*_i_ = 55.09 ± 7.58 nM, Fig. 6e). The effect of modifications at ring A (C-3), B (C-10), C (C-6, C-7, C-8), and E (C-14, tertiary nitrogen) of heroin were further investigated (Fig. 7). The 6-AmHap-Abs bound to desomorphine (*K*_i_ = 0.90 ± 0.64 nM) and thebaine (*K*_i_ = 13.08 ± 5.47 nM) (ESM Table S3 and Fig. S1). Desomorphine has no chiral center at the C6-position while thebaine has conjugated double bonds at ring C4.

For the modifications at rings B and E (ESM Table S3 and Fig. S1), 6-AmHap-Abs exhibited tight affinities to nalorphine (*K*_i_ = 2.93 ± 0.98 nM) and 10-hydroxymorphine (*K*_i_ = 18.45 ± 5.78 nM). A dramatic reduction in affinity was observed for oxymorphone (*K*_i_ = 251.07 ± 7.00 nM). Near abolition in affinity was observed for both naloxone (*K*_i_ = 1378.09 ± 146.86 nM) and morphine N-oxide (*K*_i_ = 6462.78 ± 4780.77 nM).

For ring deletion(s) (ESM Table S3 and Fig. S1), 6-AmHap-Abs exhibited tight affinity to levorphanol (*K*_i_ = 11.59 ± 4.41 nM). Significant reduction in affinity was observed for meperidine (*K*_i_ = 551.73 ± 261.76 nM). Neither acetanilide nor N-methylpiperidine bound to 6-AmHap-Abs. Binding affinities (*K*_i_) derived from MST were in the nanomolar range. IC_50_s derived from heterologous competition ELISA were in the nanomolar range while IC_50_s derived from homologous competition ELISA were in the micromolar range (ESM Table S3).

## Discussion

The measurement of antibody:antigen affinities in the presence of biological matrices (e.g., serum) by solution-based methods has long been an arduous task [13, 14, 17]. One approach to solve this problem is to use labeled antibodies, which provide the chemical properties necessary for the detection of antibody:antigen binding. In a typical setup, the antibodies are purified from sera or produced by cell culture, treated with an excess amount of tracer, and then further purified to isolate the labeled antibodies [23, 33, 34]. The exhaustive purification steps and non-specific labeling of the antibodies invariably lead to the production of labeled antibodies that have varying amounts of binding loss and/or may not reflect the binding properties of the same antibodies in their native milieu. To date, competition ED-UPLC/MS/MS is the only solution-based assay that can indirectly measure polyclonal antibody affinities without the need for antibody labeling or extensive purification due to its use of a deuterated competitor. Previously, we demonstrated competition ED-UPLC/MS/MS as a novel analytical method that can measure drug–antibody interactions [13]. Although it provides accurate *K*_d_ values for most analytes in the nanomolar range, competition ED-UPLC/MS/MS has some major drawbacks including the long hours needed for dialysis and LC/MS runs, as well as the relatively large volume of serum that is needed per experiment (5–10 μL). Alternatively, solid-based heterologous and homologous competition ELISAs require much less time to execute. Competition ELISAs, however, similarly have high sample volume requirements and have the potential to conflate antibody concentration variables with antibody affinity variables. Although the binding affinities of antibody:protein antigen that are derived from ELISA are comparable to solution-based methods [35, 36], we found that the IC_50_ values of antibody:opioid antigen interactions were platform-dependent. For example, the IC_50_s derived from heterologous ELISA were comparable to the IC_50_s derived from heterologous MST when the competitors have a relatively tight binding affinity to 6-AmHap-Abs. However, unlike heterologous MST, overestimation of IC_50_ from heterologous ELISA was observed when the competitors had moderate to weak binding affinities to the polyclonal antibodies (ESM Table S3). On the other hand, homologous ELISA tends to numerically overestimate IC_50_ by a factor of 10^3^ [13, 37] for antibody:heroin-derived molecule interactions, frequently returning IC_50_ values in the micromolar range. Collectively, homologous ELISA and homologous MST were both unsuccessful in measuring the apparent binding affinities of 6-AmHap-Abs to various opioids because they require high concentrations of the competitors to disrupt the inherent tight binding of the homologous pairs (i.e., 6-AmHap-Abs:6-AmHap ligand). It is noteworthy to mention that the overestimation of IC_50_ by homologous ELISA and by heterologous ELISA with weak competitors might only occur in ELISA platforms utilizing protein carrier-haptens with anti-hapten serum IgG. BSA is reported to be an ellipsoid with dimensions of 8 × 4 nm [38] while IgG’s antigen-binding arms are separated by a 10–15 nm spread [39–41]. Despite the fact that we have previously used a BSA-hapten coating antigen with a hapten density of three to five per protein [25], it is possible that the two arms of IgG are engaged in divalent interactions between neighboring BSA-haptens. Immobilization of the antigen in a solid support undoubtedly places protein carrier-hapten molecules within the 10–15 nm distance, which results in a high local concentration of hapten. This could therefore provide opportunity for more divalent interactions than when the antigen is in solution. It is possible that the geometry constraints of the protein carrier-hapten and IgG complex combined with the surface effects associated with solid-based ELISA [42] become more pronounced in heterologous ELISA when the competitor is weaker. In homologous ELISA, these geometry constraints and surface effects compounded with the strong association of antibody:hapten homologous pairs might drive the need for high concentrations of competitor drugs to dissociate the antibody:antigen interaction, thereby further overestimating the IC_50_ values to 10^3^-fold. These same effects may also occur with other assays, such as SPR when the protein carrier-hapten is used as the capture antigen. The shortcomings of ELISA, ED-UPLC/MS/MS, and other binding affinity assay methods thus prove that an alternative solution-based assay—offering nanomolar accuracy with a short assay time and low sample requirement—is needed.

We tested the utility of a novel heterologous MST method that we designed for our heroin vaccine development process by (1) comparing the *K*_i_ values of monoclonal ab1060 to morphine that we derived from MST and ED-UPLC/MS/MS to the values that the manufacturer derived from UV-VIS (2) comparing the *K*_i_ values of polyclonal 6-AmHap-Abs to 6-AM and morphine that we derived from both MST and ED-UPLC/MS/MS, and (3) using MST to measure the *K*_i_ values of polyclonal 6-AmHap-Abs to heroin and various other opioids and structural analogs. In doing so, we optimized a novel method that has the potential for broad-reaching applications within the field of immunology as a tool for measuring vaccine-induced unlabeled polyclonal antibody affinities to various unlabeled ligands. As expected, the *K*_i_ value of ab1060 to morphine derived from MST is consistent both with the manufacturer reported *K*_d_ value of 2 nM derived from UV-VIS [28, 29] and with the *K*_d_ value that we derived from ED-UPLC/MS/MS [13]. In contrast to ED-UPLC/MS/MS, the *K*_i_ values of ab1060 to morphine do not significantly vary when different tracer and ab1060 concentrations are used. Thus, unlike ED-UPLC/MS/MS, the MST method is anticipated to tolerate subtle variations in antibody and tracer concentrations without drastically affecting the measurement of *K*_i_ values. This means that any future assay will require fewer resources in order to optimize. In addition, the ability of ab1060 antibodies to bind morphine, MorHap-Cy5, and MorHap-acetamide, without showing any binding to Cy5 demonstrates that the binding observed in the MST experiments was a bonafide antibody:ligand interaction. The *K*_i_ values of 6-AmHap-Abs to 6-AM and morphine derived from MST were also similar to the *K*_d_ values derived from ED-UPLC/MS/MS. Collectively, we have demonstrated that MST is a reliable method for measuring antibody binding affinities with a potential for use in vaccine development.

Degradation of the target compound during its analysis is a frequent bioanalytical impediment. The psychoactive compound heroin contains two labile ester groups that are cleaved by serum esterases. For decades, the accurate analytical measurement of the binding affinity of serum antibodies to heroin has been unsuccessful due to its inherent instability. Previously, we were unsuccessful in quantifying the binding affinity of polyclonal antibody to heroin by ED-UPLC/MS/MS due to the long time needed to perform the assay [13]. We rationalized that if an assay time were short enough, and if the degradation of heroin were suppressed during its execution, it would be possible to measure the *K*_d_ of 6-AMHap-Abs to heroin accurately. We posited that the novel heterologous MST method that we developed would be successful in measuring heroin:polyclonal antibody affinity because of the shorter assay time. A typical heterologous MST assay runs for < 1 h (incubation time of antibody:tracer:competitor solution = 20 min and MST measurement time = 20 min). To address the degradation kinetics, different concentrations of heroin were subjected to buffer, and pre-immune and post-immune sera. At a fixed concentration of pre-immune serum and a varied concentration of heroin, degradation followed a first-order kinetic profile [43]. The presence of heroin-binding antibodies in post-immune sera offered a degree of protection from esterase-catalyzed degradation presumably by sequestering the drug. However, this antibody-mediated protection is only effective when the heroin concentration is less than or equal to the antibody binding site concentration. This indicates that “un-sequestered” heroin is more prone to degradation. It also suggests that there is a 1:1 binding ratio between 6-AmHap-Abs and heroin. To the best of our knowledge, the phenomenon of antibody-mediated protection of heroin from serum esterases has never been reported in the literature.

The degradation of heroin in the presence of esterase inhibitors did not follow first order kinetics, suggesting that esterase-catalyzed hydrolysis of “un-sequestered” heroin was prevented by BNPP and iso-OMPA. These organophosphorus compounds are known irreversible esterase inhibitors, which act by covalently modifying a nucleophile (e.g., serine) in the enzyme’s active site [44–46]. Using these inhibitors, heroin degradation was abolished at high (up to 4000 nM) concentrations of the drug and consequently would allow for the accurate quantification of the polyclonal antibody *K*_i_ to heroin by MST. We examined the effect of esterase inhibitors on the MST-derived binding affinity. The MST assay takes so little time to execute, however, that the addition of esterase inhibitors did not actually cause a statistically significant change to the *K*_i_ that was measured (Fig. 6b). This insignificant difference between the *K*_i_s of heroin with or without esterase inhibitors suggests that inhibitors might not be necessary for the accurate quantification of binding affinity if the assay can be executed in a short period of time. It is also possible that as small amounts of heroin degrade into 6-AM during the MST assay, the overall *K*_i_ measurement of the 6-AmHap-Abs does not change as the 6-AmHap-Abs have similar binding affinities to both compounds. Clearly, this result shows the superiority of the novel MST method over ED-UPLC/MS/MS in studying analytes which are vulnerable to esterase degradation.

Based on the binding affinity results of the polyclonal sera, 6-AmHap-Abs not only bind heroin, but also bind structurally related drugs with modifications at rings A, B, C, D, and E with varying affinities. To explain such a wide range of cross-reactivity, we refer to the crystal structures of anti-morphine antibody 9B1 in complex with morphine [47]. It is possible that the 6-AmHap-Abs have a similar binding site topology as 9B1 and it could also be argued that 6-AmHap-Abs bind the cross-reactive drugs in the same manner as 9B1 since both 9B1 and the 6-AmHap-Abs were induced by a C-3-conjugated morphine hapten. Both the topology of morphine at the 9B1-binding site and the facial recognition hypothesis [24] could be used as a basis to explain the cross-reactivity of 6-AmHap-Abs.

The facial recognition hypothesis has been used previously to explain the cross-reactivity of hapten-induced antibodies [24, 48]. In a carrier-hapten setting (e.g., TT-6-AmHap bioconjugate), the presentation of the hapten’s three-dimensional structure to the immune system is represented by two immunologically defined “faces.” The “front face” is composed of functional groups that are exposed to the immune system while the “backface” is composed of structural moieties that are sterically inaccessible to the immune system. This means that related hapten or drug structures could present similar “front faces” to the immune system and share cross-reactivity with the induced antibodies as a result. Using the facial recognition hypothesis, we anticipated that moieties at the C-3 position would constitute the back face of the 6-AmHap hapten while moieties at the C-6 position would constitute the front face. Consequently, the 6-AmHap-Abs should be able to tolerate most structural modifications at the C-3 position but be less able to tolerate structural modifications of the acetamide-like group at the C-6 position. Based on our data, however, this is not entirely the case. Based on the observed *K*_i_ values, the 6-AmHap-Abs have a comparable binding affinity to heroin, 6-AM, morphine, and morphine-6-β-glucuronide, but have a relatively lower binding affinity to morphine-3-β-glucuronide. At the C-3 position, the steric effects associated with the addition of an acetyl or hydroxy group are similar as demonstrated by the similar binding affinities of the antibodies to heroin, 6-AM, and morphine. Based on our measured *K*_i_, however, it appears that the addition of a glucuronic acid at the C-3 position has a modest impact on binding. The lowered affinity of 6-AmHap-Abs to morphine-3-β-glucuronide could be explained by the chair composition of the glucose moiety. It is possible that the chair conformation, with a charged carboxylate group, is oriented in such a way that it can fold back towards the shallow binding site, albeit at the periphery, and interfere with the hydrophobic interactions thereby reducing the binding affinity. Otherwise, the binding affinity data is consistent with morphine’s topology at the 9B1-binding site where the C-3 and C-6 positions are exposed to the aqueous milieu and are not crucial epitopes for binding. Unlike C-3, the steric effects associated with acetyl, hydroxy, and glucuronic acid at the C-6 position were all equivalently minimal. Additionally, the bulk Cy5 fluorophore at the C-6 position did not seem to drastically impact the binding of 6-AmHap-Abs to MorHap-Cy5. It is noteworthy to mention that 6-AmHap-Abs were generated using a hapten with an S configuration at the C-6 position and with no double bond at ring C. In contrast, the MorHap-Cy5 tracer has an opposite chirality at C-6 (i.e., R configuration) and a double bond at ring C. The ability of 6-AmHap-Abs to bind MorHap-Cy5 suggests that the mismatched chirality at C-6 and the unsaturation at ring C are tolerated well by the antibodies. The stereochemistry at the C-6 position is not important for binding as seen in the ability of 6-AmHap-Abs to bind desomorphine and thebaine, neither of which have a chiral center at the C-6 position. This implies that 6-AmHap-Abs are permissive to structural modifications at the C-6 position. Despite the binding affinity result of morphine-3-β-glucuronide, which was only modestly different than expected, the facial recognition hypothesis holds true as 6-AmHap-Abs were able to tolerate all other modifications at the C-3 and C-6 positions very well, and were able to bind heroin, 6-AM, morphine, morphine-3-β-glucuronide, morphine-6-β-glucuronide, desomorphine, and thebaine, just as the hypothesis would predict.

To probe the effect of substituents at the putative front face, composed of rings B and E, the binding affinities of 6-AmHap-Abs to 10-hydroxymorphine, oxymorphone, nalorphine, naloxone, normorphine, and morphine N-oxide were evaluated. The effect of substituting a hydroxy group at different positions revealed an interesting trend. The placement of the hydroxy group at the C-10 position, as in 10-hydroxymorphine, had minimal effect on 6-AmHap antibody binding, while placement at the C-14 position, as in oxymorphone, dramatically reduced binding. As discussed vide supra, the effects of the double bond at ring C and the substituent at the C-6 position have minimal impact on binding, and thus, the poor binding of 6-AmHap-Abs to oxymorphone is solely attributable to the hydroxy group at the C-14 position. There are three possible effects that this hydroxy group has on binding. First, it is possible that there are no hydrogen bond acceptors in the binding site that can complement with the hydroxy group. Second, it is possible that the C-14 hydroxy group and the tertiary nitrogen compete for the same acidic residue at the binding site. The formation of an ion–dipole interaction between the C-14 hydroxy group and an acidic residue would result in the presence of a positive charge in a predominantly hydrophobic environment: a thermodynamically unfavorable state [49–51]. Third, the hydroxy group may create steric hindrances in the binding site thereby preventing the close packing of the oxymorphone with the binding site’s residues [52, 53]. The effect of the C-10 hydroxy group in 10-hydroxymorphine on the reduction of 6-AmHap antibody binding affinity is not as dramatic as the effect the C-14 hydroxy group has presumably because the C-10 hydroxy group is pointed away from the front face and does not cause steric clashes with crucial binding residues.

The lengthening of the methyl group (one carbon) into a propylene group (three carbons) on the tertiary nitrogen in nalorphine did not reduce the binding affinity. This suggests that the additional three carbons do not result in unfavorable steric crowding. However, the presence of a C-14 hydroxy group in addition to a propylene group on the tertiary nitrogen, as in naloxone, nearly abolished the binding of 6-AmHap-Abs altogether. The cumulative steric effects of these two groups presumably affect the packing of the naloxone into the binding site and possibly even change the arrangement of side chains at the binding site.

The stepwise deletion of heroin’s ring system provides further insight into the characteristics of 6-AmHap antibody:opioid binding interactions. The deletion of ring D had a minimal effect on the binding of 6-AmHap-Abs to levorphanol. The simultaneous deletion of rings B, C, and D in meperidine, however, dramatically reduced the binding of 6-AmHap-Abs. This was despite the fact that with respect to atom economy, meperidine is comparable to levorphanol. Due to the deletion of the ring systems, meperidine is highly flexible. It is possible that these ring systems function to properly orient the crucial epitopes of the hapten within the binding site by constraining the conformational flexibility of rings A and E. Since binding affinity is governed both by Gibb’s free energy and entropy [54], we speculate that the favorable cation-π and salt-bridge interactions between the 6-AmHap-Ab binding site and meperidine are being offset by the entropic penalty of orienting the meperidine molecule at the binding site. Isolated ring A (acetanilide) and ring E (N-methylpiperidine) did not bind 6-AmHap-Abs possibly due to insufficient molecular interactions between the 6-AmHap-Abs and these drugs or perhaps due to improper alignment in the binding site. By using the novel heterologous MST method that we developed, we were able to dissect the epitopes of heroin which are crucial in order for 6-AmHap-Abs to bind. The dissection of the 6-AmHap-Ab cross-reactivity would have not been possible if we had employed ED-UPLC/MS/MS because of the limited amount of sera available. The binding affinity data derived from MST revealed that the cross-reactivity of the 6-AmHap-Abs stems from the antibodies’ tolerance of modifications to key epitopes of the hapten. In addition, the results are consistent with the facial recognition hypothesis. To the extent of our knowledge, a comprehensive discussion on heroin epitope:antibody interactions has not been presented in the literature. The structure:antibody affinity relationship presented here would therefore be useful as guidance for future opiate hapten design efforts.

Despite the ability of MST to measure a wide range of binding affinities in polyclonal sera, the assay does have four major limitations. First, in all types of MST, the concentration of the antibodies in the serum must be known. The concentration of the antibodies and the binding affinity can be inferred from homologous MST, though this does require extensive and complex mathematical calculations [16]. Alternatively, the concentration of antibody binding sites can be determined by ED-UPLC/MS/MS, but this makes conventional and heterologous MST experiments dependent on an assay that, in and of itself, measures binding affinities. The fact that researchers can subsequently perform countless MST experiments using this concentration data, however, offsets this downside. Second, the fluorescent tracer must only have a modest binding affinity to the polyclonal antibodies. Heterologous MST would not be possible or would only be possible for a limited number of competitors if the affinity of the fluorescent tracer to the antibody was too high or too low. Third, the determination of the *K*_d_ for the polyclonal antibody:ligand binding needs to be done at a relatively high serum dilution since MST is sensitive to changes in buffer composition. At low serum dilutions, a change in fluorescent signal can arise from non-binding events because of the drastic change in the solvent composition. This can consequently produce artifactual signals that affect the calculation of the *K*_d_. Fourth, although the synthesis of MorHap is straightforward [26], the preparation of a cross-reactive hapten to a new ligand/analyte might be challenging depending on the hapten structure and should thus be a major consideration prior to the execution of heterologous MST.

Despite these limitations, MST is a simple and fast method for determining binding affinities with a low sample requirement. After the initial investment on the MST apparatus, the MST assay itself is relatively inexpensive to perform. Since the MST method is straightforward and does not require a high level of expertise, MST assays can be performed routinely in the laboratory. In addition, the measurement of binding affinities by MST takes less than 1 h, allowing for the daily execution of multiple binding experiments including for analytes which are prone to rapid degradation. A typical heterologous MST experiment only requires ~ 1 μL of polyclonal serum. Thus, the MST assay becomes indispensable when a limited amount of serum is available despite needing to test against numerous drug competitors. Overall, our novel heterologous MST method can be used to measure a wide range of *K*_i_ values and has the potential to perform high-throughput binding affinity screenings of polyclonal sera.

## Electronic supplementary material

ESM 1 (PDF 480 kb)
