# Supplementary material for: A rapid solution-based method for determining the affinity of heroin hapten-induced antibodies to heroin, its metabolites, and other opioids
Source: Anal Bioanal Chem. 2018 Apr 19;410(16):3885–903. doi: 10.1007/s00216-018-1060-4 (PMC5956019; doi:10.1007/s00216-018-1060-4)
Supplement: Supplementary file 1 — (PDF 480 kb) [file 216_2018_1060_MOESM1_ESM.pdf]

Analytical and Bioanalytical Chemistry

Electronic Supplementary Material

A rapid solution-based method for determining the affinity of heroin hapten-induced antibodies to heroin, its metabolites, and other opioids

Oscar B. Torres, Alexander J. Duval, Agnieszka Sulima, Joshua F. G. Antoline, Arthur E. Jacobson, Kenner C. Rice, Carl R. Alving, Gary R. Matyas

INDEX	PAGE
Synthesis of Cy5 Negative Control Tracer	S3
Synthesis and Analytical Characterization of **1**, **2**, **3**, and MorHap-acetamide	S3-S6
Heterologous Competition ELISA	S6
**Table S1** IC_{50} and K_i of ab1060 to morphine at different concentrations of ab1060 and MorHap-Cy5 tracer derived from MST	S7
**Table S2** Dissociation constants of 6-AmHap-Abs against 6-AM and morphine derived from ED-UPLC/MS/MS	S8
**Table S3** IC_{50} and K_i of 6-AmHap-Abs to various drug competitors derived from competition ELISA and MST	S9
**Fig. S1** MST Competition binding curves of 6-AmHap-Abs to various opioid competitors	S10
**References**	S11

Synthesis of Cy5 Negative Control Tracer

Cy5 was used as a negative control for testing the binding of both monoclonal antibody ab1060 and polyclonal 6-AmHap-Abs. Sulfo-Cyanine5 NHS ester was purchased from Lumiprobe Corporation (Hallandale Beach, Florida, USA) and dissolved in DPBS. The mixture was allowed to hydrolyze overnight on a stir plate and was subsequently purified on an XBridge® BEH C4 OBD prep column, 300 Å, 5 µM, 19 mm x 150 mm, and lyophilized to yield a dark blue powder. The tracer was characterized by MALDI-TOF MS ($M+H^+$= 642.24 m/z).

Synthesis and Analytical Characterization of 1, 2, 3, and MorHap-acetamide

ACS reagent grade chemical reagents, such as imidazole, *tert*-butyldimethylsilyl chloride, triethylamine, 2-bromoacetamide, triethylsilane, cesium fluoride, and solvents such as anhydrous tetrahydrofuran (THF), chloroform, and dimethylformamide (DMF) that were used in organic synthesis were purchased from Sigma-Aldrich (Saint Louis, MO). All melting points were determined on a Thomas-Hoover meltingpoint apparatus and are uncorrected. Proton nuclear magnetic resonance (^{1}H NMR, 500 MHz) and carbon nuclear magnetic resonance (^{13}C NMR, 100 MHz) spectra were recorded on a Bruker 500 instrument in $CDCl_3$ (unless otherwise noted) with the values given in ppm (TMS as internal standard) and J (Hz) assignments of ^{1}H resonance coupling. The high resolution mass spectra (HRMS) were recorded using electrospray ionization (ESI) on a Waters LCT Premier time-of-flight (TOF) mass spectrometer. Thin Layer chromatography (TLC) was performed on 0.25 mm Analtech GHLF silica gel. Flash column chromatography was performed with Bodman silica gel LC 60 A. Units for $[\alpha]_D$ values were given in 10^{-1} deg cm^2 g^{-2}.

***N*-((4a*R*,7*R*,7a*R*,12b*S*)-9-((*tert*-Butyldiphenylsilyl)oxy)-3-methyl-2,3,4,4a,7,7a-hexahydro-1*H*-4,12-methanobenzofuro[3,2-*e*]isoquinolin-7-yl)-3-(tritylthio)propanamide, 1**. MorHap (0.19 g, 0.31 mmol) was dissolved in chloroform (5 mL) under N_2, and imidazole (0.084 g, 1.24 mmol, 4 equiv) and *tert*-butyldimethylsilyl chloride (0.17 g, 0.62 mmol, 0.16 mL, 2 equiv) were added sequentially. The solution was heated to 50 °C until the starting material was consumed by TLC. The solution was cooled, washed with water (3 x 5 mL), dried over Na_2SO_4, filtered, and concentrated to give a yellow oil. The oil was purified via column chromatography on SiO_2 (99:0.9:0.1 $CHCl_3$:MeOH:NH_4OH) to give a white foam (0.187 g, 70%).

^{1}H-NMR (400 MHz; $CDCl_3$): δ 7.74-7.68 (m, 4H), 7.44-7.19 (m, 20H), 6.39 (d, J = 8.1 Hz, 1H), 6.23 (d, J = 8.1 Hz, 1H), 5.66 (ddd, J = 9.6, 6.1, 3.2 Hz, 1H), 5.52 (dd, J = 9.6, 1.3 Hz, 1H), 5.05 (d, J = 7.1 Hz, 1H), 4.60 (s, 1H), 4.07 (t, J = 6.5 Hz, 1H), 3.26 (dd, J = 5.4, 3.2 Hz, 1H), 2.92 (d, J = 18.5 Hz, 1H), 2.86 (s, 1H), 2.51 (m, J = 6.7 Hz, 3H), 2.39 (s, 3H), 2.30-2.18 (m, 2H), 1.97 (t, J = 7.1 Hz, 2H), 1.91 (dt, J = 12.4 Hz, 1H), 1.65 (d, J = 11.2 Hz, 1H), 1.10 (s, 9H); ^{13}C NMR (100 MHz; $CDCl_3$): δ 170.5, 148.0, 144.8, 137.5, 135.83, 135.77, 133.9, 133.4, 133.0, 130.5, 129.7, 129.0, 128.1, 127.62, 127.60, 127.43, 126.8, 120.7, 118.6, 92.0, 67.0, 59.1, 49.6, 47.0, 44.2, 43.2, 40.4, 36.0, 35.8, 27.9, 26.84, 26.77, 20.4, 19.8; HRMS (ESI+) (*m/z*) calc'd for $C_{55}H_{57}N_2O_3Si$ 853.3859, found 853.3850; anal calc'd for $C_{55.5}H_{56.5}Cl_{1.5}N_2O_3SSi$ [M + 0.5 $CHCl_3$] C, 73.02; H, 6.24; N, 3.07; found C, 73.15; H, 6.09; N, 3.07.

3-((2-Amino-2-oxoethyl)thio)-*N*-((4a*R*,7*R*,7a*R*,12b*S*)-9-((*tert*-butyldiphenylsilyl)oxy)-3-methyl-2,3,4,4a,7,7a-hexahydro-1*H*-4,12-methanobenzofuro[3,2-*e*]isoquinolin-7-yl)propanamide, 3. To a solution of amide **1** (0.17 g, 0.20 mmol) in chloroform (4.0 mL) in a scintillation vial under N_2 equipped with a stir bar was added triethylsilane (0.100 mL, 2.5% v/v) and trifluoroacetic acid (0.40 mL, 10% v/v). The solution was stirred at 25 °C until the starting material was consumed as indicated by TLC analysis, ca. 30 min. The solution was concentrated at 0 °C *in vacuo* for 2 h to give a white solid **2**. The solid was dissolved in DMF (4.0 mL) and triethylamine (0.12 g, 1.20 mmol, 0.170 mL, 6 equiv) and 2-bromoacetamide (0.055 g, 0.40 mmol, 2 equiv) were added sequentially. The solution was stirred at 25 °C for 16 h then concentrated *in vacuo* to give a yellow oil. The oil was purified via column chromatography on SiO_2 (98:1.8:0.2 to 90:9:1 $CHCl_3$:MeOH:NH_4OH) to give amide **3** as a yellow foam (0.086 g, 65%).

3-((2-Amino-2-oxoethyl)thio)-*N*-((4a*R*,7*R*,7a*R*,12b*S*)-9-hydroxy-3-methyl-2,3,4,4a,7,7a-hexahydro-1*H*-4,12-methanobenzofuro[3,2-*e*]isoquinolin-7-yl)propanamide, MorHap-acetamide. To a solution of **3** (0.051 g, .073 mmol) in 1:1 MeOH:$CHCl_3$ (2 mL) was added cesium fluoride (0.069 g, 0.46 mmol, 6 equiv). The solution was stirred for 16 h at 25 °C, wherein the starting material was consumed as indicated by TLC. The solution was concentrated *in vacuo* and purified via column chromatography on SiO_2 (95:4.5:5 to 88:10.8:1.2 $CHCl_3$:MeOH:NH_4OH) to give MorHap-acetamide as a clear oil (0.024 g, 73%). The oil was lyophilized in 15:1 *tert*-butanol:MeOH to give an off-white solid.

^{1}H-NMR (400 MHz; CD_3OD): δ 6.54 (d, *J* = 8.1 Hz, 1H), 6.46 (d, *J* = 8.1 Hz, 1H), 5.73 (ddd, *J* = 9.5, 5.6, 3.3 Hz, 1H), 5.66 (d, *J* = 10.0 Hz, 1H), 4.63 (s, 1H), 4.31 (d, *J* = 5.6 Hz, 1H), 3.37 (dd, *J* = 5.4, 3.4 Hz, 1H), 3.22 (s, 2H), 3.03 (d, *J* = 18.8 Hz, 2H), 2.91-2.88 (m, 2H), 2.62-2.50 (m, 3H), 2.45 (s, 3H), 2.41-2.34 (m, 2H), 2.05 (td, *J* = 12.6, 4.9 Hz, 1H), 1.72 (dd, *J* = 12.6, 2.2 Hz, 1H); ^{13}C NMR (100 MHz; CD_3OD): δ 175.3, 173.7, 145.8, 140.3, 133.0, 131.2, 129.3, 126.6, 120.3, 118.0, 94.0, 60.5, 51.4, 48.2, 45.1, 43.0, 40.4, 36.5, 36.3, 35.9, 29.7, 21.3; HRMS (ESI+) (*m/z*) calc'd for $C_{22}H_{28}N_3O_4S$ 430.1801, found 430.1796.

Heterologous Competition ELISA

Homologous competition ELISA (coating antigen: BSA-6-AmHap conjugate) was previously reported by Sulima et al [3]. Heterologous competition ELISA was performed as described [3] except that BSA-MorHap was used as a coating agent. Coating antigens with similar hapten densities (~4) and concentrations (0.1 µg/well; 0.1 mL of 1 µg/mL antigen) were used in both homologous and heterologous competition ELISA. The drug competitors were diluted in log order concentrations in microtiter plates and mixed with diluted sera to give final competitor concentrations between 0.1 and 10,000 nM.

Table S1 IC_{50} and K_i of ab1060 to morphine at different concentrations of ab1060 and MorHap-Cy5 tracer

[MorHap-Cy5], nM	[Ab1060], nM	IC_{50}, nM[a]	K_i, nM[b,c]
0.25	5	9.41 ± 1.50	3.13 ± 0.49
	10	28.20 ± 1.00	6.87 ± 2.99
	20	50.32 ± 1.9	6.87 ± 2.95
	30	70.03 ± 1.38	5.91 ± 1.49
	40	77.67 ± 1.58	4.17 ± 0.91
0.5	5	14.15 ± 0.82	5.63 ± 2.84
	10	26.23 ± 0.93	5.95 ± 1.14
	20	37.74 ± 1.45	4.18 ± 2.73
	30	56.52 ± 1.06	4.37 ± 3.13
	40	70.33 ± 1.50	3.53 ± 1.60
1.0	5	15.70 ± 0.72	5.79 ± 0.62
	10	19.50 ± 0.97	3.82 ± 1.19
	20	43.81 ± 0.88	5.32 ± 2.16
	30	55.38 ± 1.72	4.11 ± 1.90
	40	99.24 ± 1.85	6.60 ± 2.20

[a] – Measured with a starting morphine concentration of 4,000 nM.

[b] – K_i calculated using K_d of 4.58 ± 2.19 nM.

[c] – K_i values were not statistically different by ANOVA.

Table S2 Dissociation constants of 6-AmHap-Abs against 6-AM and morphine derived from ED-UPLC/MS/MS

Drugs	b values[a]	K_d, nM[b]	$[Ab_t]$, nM[c]	[Antibodies], nM[d,e]
6-AM	0.61 ± 0.02	0.53 ± 0.28	1.58 ± 0.44	2527.92 ± 704.93
Morphine	0.56 ± 0.03	0.51 ± 0.19	1.36 ± 0.25	2172.06 ± 397.96

[a] −All b were calculated using the equation provided by Torres et al. [1] and are mean of triplicate determinations ± standard deviation.
[b] −All K_d values were calculated using Müller's equation [1,2] and are mean of triplicate determinations ± standard deviation. The $[T_t]$ is 1.25 nM.
[c] – The $[Ab_t]$ was calculated using the b value and K_d in the equation described in the methods section of the main text.
[d] –The [Antibodies] in the undiluted post-immune serum were calculated by multiplying $[Ab_t]$ by serum dilution (1600). The main assumption is that there was a negligible change in the volume of buffer in the sample chamber during ED because of the sealing tape on the ED plate.
[e] –The [6-AmHap-Abs] was calculated by averaging [6-AM binding antibodies] and [Morphine binding antibodies]. The [6-AmHap-Abs] in the undiluted post-immune serum was calculated to be 2350 nM.

Table S3 IC_{50} and K_i of 6-AmHap-Abs to various drug competitors derived from competition ELISA and MST

Competitor	Competition ELISA Homologous, IC_{50}, µM[a]	Competition ELISA Heterologous, IC_{50}, nM	Heterologous MST IC_{50}, nM	Heterologous MST K_i, nM[b]
**Heroin**				
Heroin w/ Esterase Inhibitor	N/A[d]	N/A	11.19 ± 1.03	1.60 ± 0.75
Heroin w/o Esterase Inhibitor	1.27 ± 0.3	11.10 ± 3.85	10.29 ± 1.32	2.27 ± 0.95
**Rings A and C**[c]				
6-AM	3.72 ± 0.7	19.75 ± 6.33	6.75 ± 1.17	0.44 ± 0.33
M-6G	24.98 ±12.1	11.29 ± 2.16	5.88 ± 1.22	0.66 ± 0.90
Desomorphine	N/A	N/A	9.07 ± 0.73	0.90 ± 0.64
Morphine	2.36 ± 0.6	7.50 ± 1.93	8.40 ± 0.95	1.00 ± 0.66
Thebaine	N/A	N/A	35.61 ± 0.91	13.08 ± 5.47
M-3G	27.98 ± 11.4	23.21 ± 7.33	69.10 ± 1.24	27.92 ± 4.14
**Rings B and E**[c]				
Nalorphine	N/A	N/A	10.59 ± 0.89	2.93 ± 0.98
10-Hydroxymorphine	N/A	N/A	51.21 ± 0.72	18.45 ± 5.78
Normorphine	112.43 ± 22.3	3224.56 ± 2002.34	135.97 ± 0.94	55.09 ± 7.58
Oxymorphone	125.49 ± 28.6	2431.22 ± 864.44	598.14 ± 0.95	251.07 ± 7.00
Naloxone	>1000	2047.25 ± 1288.66	3037.8 ± 1.37	1378.09 ± 146.86
Morphine N-Oxide	N/A	N/A	12651 ± 1.33	6462.78 ± 4780.77
**Ring Deletions**[c]				
Levorphanol	3.39 ± 0.9	340.77 ± 138.46	33.69 ± 0.91	11.59 ± 4.41
Meperidine	523.89 ± 143.3	7025.78 ± 6058.72	1172.3 ± 1.13	551.73 ± 261.76
N-Methylpiperidine	N/A	N/A	No binding[e]	No binding
Acetanilide	N/A	N/A	No binding	No binding

[a] – Values from Sulima et al [3].
[b] – K_i calculated using K_d of 7.65 ± 0.86 nM
[c] – Type of modification in the heroin's ring system.
[d] – Not applicable. The experiment was not performed.
[e] – No binding. The experiment was performed, but no binding was observed at any of the drug concentrations tested.

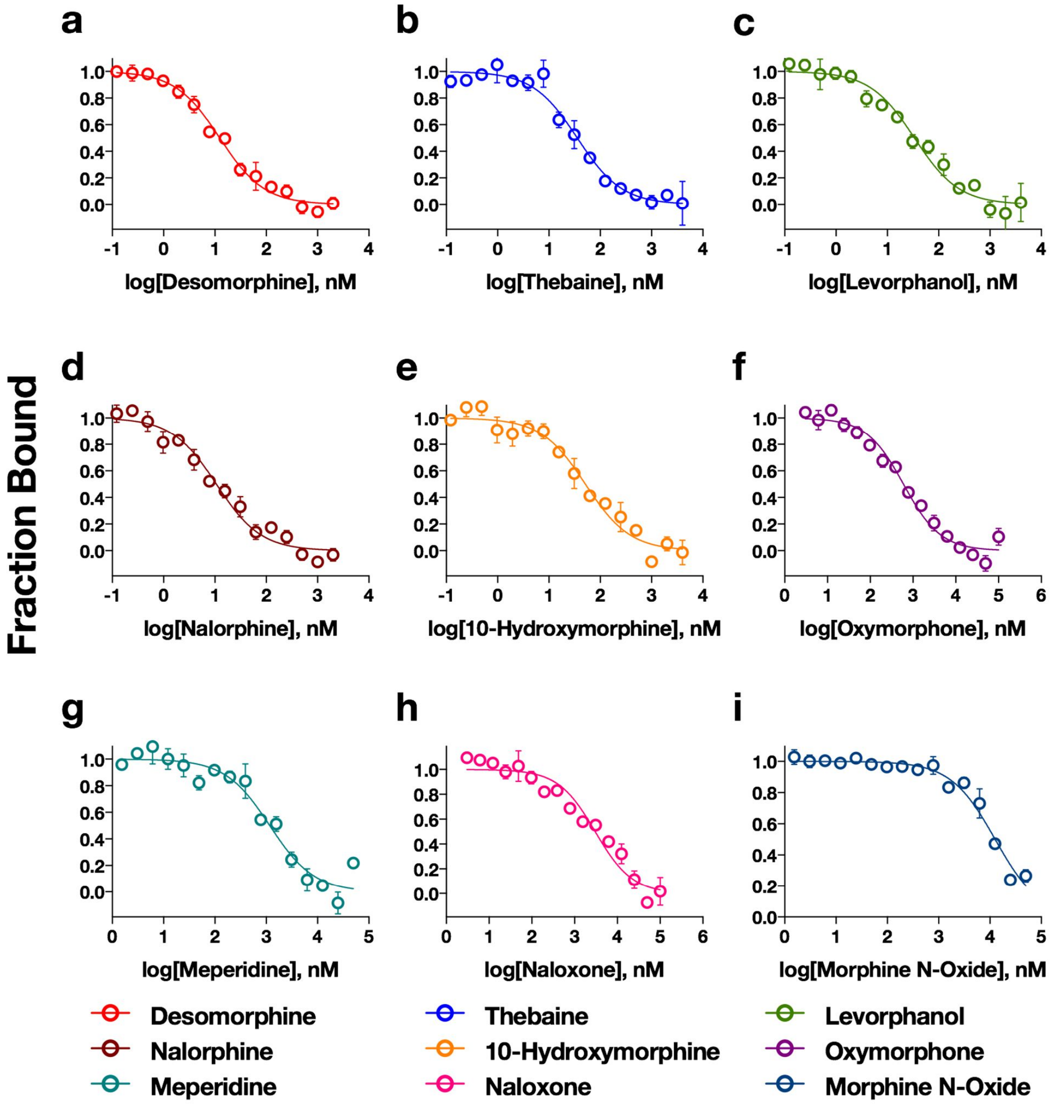

Fig. S1 MST Competition binding curves of 6-AmHap-Abs to various opioid competitors. The opioid competitors were: desomorphine (**a**), thebaine (**b**), levorphanol (**c**), nalorphine (**d**), 10-hydroxymorphine (**e**), oxymorphone (**f**), meperidine (**g**), naloxone (h), and morphine N-oxide (**i**). The MST Competition binding curves were arranged in decreasing binding affinities. The binding curves are derived from 3 independent experiments ± standard error of the mean

References

1. Torres OB, Antoline JF, Li F, Jalah R, Jacobson AE, Rice KC et al. A simple nonradioactive method for the determination of the binding affinities of antibodies induced by hapten bioconjugates for drugs of abuse. Anal Bioanal Chem. 2016;408(4):1191-204. doi:10.1007/s00216-015-9223-z.

2. Müller R. Determination of affinity and specificity of anti-hapten antibodies by competitive radioimmunoassay. Methods Enzymol. 1983;92:589-601.

3. Sulima A, Jalah R, Antoline JF, Torres OB, Imler GH, Deschamps JR et al. A Stable Heroin Analog That Can Serve as a Vaccine Hapten to Induce Antibodies that Block Effects of Heroin and its Metabolites in Rodents and that Cross-React Immunologically with Related Drugs of Abuse. J Med Chem. 2018;61(1):329-43. doi:10.1021/acs.jmedchem.7b01427.
